# C_20_ and nitrogen-substituted fullerenes: anharmonic IR and UV-vis spectra for astrophysical environments

**DOI:** 10.1039/d5ra05271h

**Published:** 2025-10-28

**Authors:** Venkata Lakshmi Karri, Ajay Chaudhari, Takashi Onaka, Mahadevappa Naganathappa

**Affiliations:** a Department of Physics, School of Science, GITAM (Deemed to be University) Hyderabad 502329 TS India swamimahadev25@gmail.com; b Department of Physics, The Institute of Science, Dr Homi Bhabha State University Madame Kama Road, FORT Mumbai 400032 MH India; c Department of Astronomy, Graduate School of Science, The University of Tokyo 7-3-1 Hongo Tokyo 113-0033 Japan

## Abstract

Theoretical infrared (IR) and electronic absorption spectra of the C_20_ fullerene and its nitrogen-substituted heterofullerenes in gas and water solvent are studied and discussed in terms of astronomical observations. The replacement of a carbon atom by nitrogen results in two stable heterofullerenes, which is confirmed by their HOMO to LUMO energy gap. The ionization potential and electron affinity of these molecules are reported. Theoretical calculations performed at the B3LYP/6-311++G(d,p) level of density functional theory (DFT). The effect of water solvent is studied using the integral equation formalism polarized continuum model (IEFPCM) at the same level of theory. Effects of substitution on the electronic and absorption spectra of these molecules are studied. The results of the C_20_ fullerene and its heterofullerenes show spectra with peaks at 6.2, 6.67, 7.0, 7.7, 8.5, 11.3, and 12.8 μm, which have corresponding features in observed spectra of the planetary nebulae Tc1 and NGC 7027, and the reflection nebulae NGC 2023 and NGC 7023. The electronic absorption spectra of these molecules are also calculated by time-dependent DFT (TD-DFT) and discussed in relation to the ultraviolet bump feature at 217 nm in the interstellar extinction curve. We estimate the transition wavelength, oscillator strength, and symmetry using the AOMix program.

## Abstract 2

Theoretical infrared (IR) and electronic absorption spectra of the C_20_ fullerene and its nitrogen-substituted heterofullerenes in gas and water solvent are studied and discussed in terms of astronomical observations.

## Graphical abstract

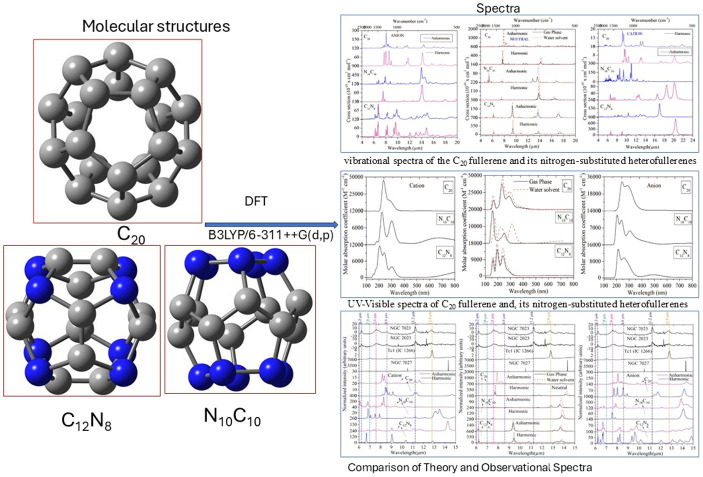

## 1 Introduction

Infrared (IR) spectra of various astronomical objects, from circumstellar environments and the interstellar medium of our galaxy to external galaxies, exhibit strong emission features at 3.3, 6.2, 7.7, 8.6, and 11.2 μm.^1,2^ These are attributed to large carbonaceous molecules composed of benzene rings, such as polycyclic aromatic hydrocarbons (PAHs).^3–5^ Another class of carbonaceous materials, fullerenes, is also responsible for several features in IR spectra.^6–14^ PAHs have planar structures of fused benzene rings, whereas fullerenes form closed carbon cages.

The discovery of C_60_ and C_60_^+^ in laboratory experiments^15^ and the development of synthesis methods for C_60_ and C_70_ (ref. 16) paved the way for their astrophysical identification. Cami *et al.*^6^ reported the first IR detection of C_60_ and weak features of C_70_ in the planetary nebula Tc1. Since then, features of fullerenes have been identified in various environments such as planetary nebulae,^17,18^ reflection nebulae,^19,20^ proto-planetary nebulae,^21^ and young stellar objects.^22^ The bands observed in NGC 7023 at 6.4, 7.1, 8.2, and 10.5 μm are attributed to C_60_^+^.^23^ IR features near 6.6, 9.8, and 20 μm have been reported in both galactic and extragalactic planetary nebulae^18,24^ and Sgr B2,^25^ resembling spectra of planar C_24_.^26^ C_24_ has also been suggested as a carrier of the 11.2 μm band in NGC 7027.^27^ The detection of both large (C_60_ and C_70_) and small (C_24_) fullerenes has broadened the understanding of carbonaceous chemistry in evolved stars.^15^

Several theoretical studies have focused on small or modified fullerenes. Adjizian *et al.*^28^ proposed that unassigned IR bands could arise from small fullerenes and modelled the IR spectra of C_20_ to C_60_ in various charge states using Density Functional Theory (DFT) calculations. Gómez-Muñoz *et al.*^29^ suggested that hydrogenated amorphous carbon grains formed in planetary nebulae could carry the 12 μm plateau. Fulleranes^30^ and nitrogen-doped fullerenes^31^ have also been studied in the context of IR and ultraviolet (UV) spectral features and chemical stability. Foing and Ehrenfreund first proposed that the Diffuse Interstellar Bands (DIBs) at 958 and 963 nm could be due to C_60_^+^.^32^ Laboratory confirmation^33^ and observational studies^34–36^ supported this proposal. Other DIBs have also been attributed to C_60_^+^ (ref. 37) and C_70_^+^.^38^ Iglesias-Groth *et al.*^39^ reported IR emission bands, which can be attributed to neutral, cationic, and anionic fullerenes in the IC 348 star-forming region. The cationic C_60_ was found to emit strongly at 11.21, 16.40, and 20–21 μm, in addition to the well-known 17.4 and 18.9 μm bands.^40^ Further studies investigated fullerene cage stability and astrochemical reactivity.^41,42^ The UV bump at 217.5 nm seen in the interstellar extinction curve—attributed to π → π* transitions in sp^2^ carbon systems—has been associated with PAHs and other carbonaceous materials.^43–46^ While PAHs are major contributors^47,48^ fullerene species may also play a role.^26,49,50^

Theoretical investigations, even before the first detection in space, explored the geometry and electronic structures of fullerenes across a wide size range from C_20_ to C_720_.^51,52^ C_20_ is the smallest fullerene, with a strained dodecahedral cage composed of 12 pentagons, lacking pentagon isolation and thus being less stable than C_60_.^53,54^ Alternative isomers, such as rings and chains, have also been studied.^55–57^ C_20_ was synthesized from dodecahedrane *via* debromination^58,59^ with ion beam irradiation.^60^ Its IR and UV spectra have been modelled.^61–63^

Substitution of carbon atoms with heteroatoms like nitrogen, boron, or oxygen yields heterofullerenes.^64,65^ The first nitrogen-substituted fullerene was identified using mass spectrometry.^66,67^ Li–fullerene interactions have also been studied,^68^ supporting stable ion-cage complexes. Theoretical investigations^27,69^ indicate that nitrogen substitution in small fullerenes such as C_20_ enhances their stability and modifies their electronic structure, making them more relevant for astrophysical environments than the pristine C_20_ cage. These results emphasize the importance of the study of small fullerenes with nitrogen substitution. Those small cyclic hydrocarbon species may be formed *via* reactions on the surface of dust grains with an ice mantle.^70^ Therefore, it is also of interest to study the effect of ice mantle on the spectroscopic properties of C_20_.

Fullerenes were first predicted theoretically and later confirmed experimentally. However, laboratory identification of fullerene species remains difficult due to challenges in studying them in an isolated condition. Thus, theoretical approaches remain essential for understanding and predicting their properties. Small fullerenes are generally less stable than larger ones, such as C_60_ and C_70_, and their detection is further complicated when they are part of a complex mixture of fullerenes, consisting of various sizes, charge states, and possible substitutions. These result in overlapping or weak spectroscopic features that hinder clear identification. The discovery of C_60_, C_60_^+^, and C_70_ fullerenes in interstellar and circumstellar environments suggests possibilities for the presence of other fullerenes and their derivatives. Identifying fullerenes in various astronomical environments relies on spectroscopic data analysis, which requires both laboratory and theoretical studies.

The present study investigates the vibrational and electronic spectroscopic properties of the C_20_ fullerene and its nitrogen-substituted derivatives (N_10_C_10_ and C_12_N_8_) in neutral, cationic, and anionic states using DFT. To simulate astrophysical environments, we also consider the spectra of C_20_ in a water solvent environment as an approximation of ice mantle conditions. While earlier studies employed harmonic DFT methods to model small fullerene IR spectra,^24^ this work presents-for the first time-anharmonic DFT calculations for both pristine and nitrogen-substituted C_20_ fullerenes across various charge states. In addition to IR spectra, we compute near-UV-visible absorption spectra to assess their potential contribution to the prominent 217.5 nm UV extinction bump. By combining charge state, nitrogen substitution symmetry, and solvent effects, this study provides a comprehensive understanding of the spectroscopic behavior and astrophysical relevance of small fullerenes. Recent radio observations have detected faint emission lines from nitrogen-containing small PAHs and cyclic hydrocarbons, suggesting that nitrogen is commonly found in interstellar carbon-based molecules.^70–79^ This supports the idea that nitrogen-substituted fullerenes may also exist in space. Including water as a solvent in our calculations helps us understand the effects of interstellar ices or polar environments on their infrared spectra.

## 2 Computational details

All the theoretical calculations for C_20_ and its nitrogen-substituted heterofullerenes (N_10_C_10_ and C_12_N_8_) are performed using the Gaussian 16 software package.^80^ An *et al.* and Gianturco *et al.*^81,82^ theoretically studied the molecular properties of C_20_ isomers in their neutral and anionic forms using various methods and basis sets. They confirmed that B3LYP/cc-pVTZ predicts the most accurate results for both the neutral and anionic low-lying isomers of C_20_. Saito *et al.*^83^ conducted their study at the B3LYP/6-311+G(d) level of theory, while Soleimani Amiri *et al.*^69^ used the B3LYP/aug-cc-pVTZ level of theory. In the present study, we optimized C_20_ molecules using the HF, B3LYP, MP2, and CCSD methods with 6-311++G(d,p), TZVP, aug-cc-pvtz, aug-cc-pvdz, and aug-cc-pvqz. Of these, the hybrid functional method B3LYP with the TZVP, aug-cc-pVTZ, and 6-311++G(d,p) basis sets is predicted to have the lowest energy for C_20_, respectively. Among these three basis sets, the variation in the obtained lowest energy is minimal, with the differences only in the decimal range, on the order of 0.01–0.02 eV. Taking into account a balance between accuracy and computational cost for both IR and UV spectra, we employ the B3LYP/6-311++G(d,p) level of theory in the present study. This level of theory is used to calculate the vibrational frequencies and electronic absorption spectra of the studied structures in their neutral, cationic, anionic, and water-solvated states. The B3LYP/6-311++G(d,p) approach has been extensively applied to PAH vibrational studies and has been shown to reliably reproduce experimental spectra.^84,85^ Although more recent hybrid functionals such as M06-2X and ωB97XD may provide improved accuracy for certain systems,^86,87^ benchmarking studies indicate that B3LYP remains a well-validated and widely accepted choice for PAHs and related carbon clusters, offering a good compromise between accuracy and efficiency. We use the integral equation formalism polarized continuum model (IEFPCM)^88–90^ with the dielectric constant of 78.4 (ref. 91) at the same level of theory to calculate IR and UV absorption spectra of water solvent states. The Cartesian coordinates of all optimized structures in their neutral, cationic, and anionic forms under both gas and solvent phases are provided in the SI (Table S2, SI).

In the present study, anharmonic vibrational spectra of the C_20_ fullerene and its nitrogen-substituted derivatives (N_10_C_10_ and C_12_N_8_) are computed using the Gaussian 16 software with the deperturbed second-order vibrational perturbation theory (DVPT2) method.^92^ This approach accounts for higher-order force constants, quadratic, cubic, and quartic terms, in the potential energy surface, enabling a more accurate representation of molecular vibrations. While a scaling factor of 0.9613 (ref. 93) is applied to harmonic frequencies to compare with experimental spectra, no such correction is needed for anharmonic calculations. We select N_10_C_10_ and C_12_N_8_ as representative nitrogen-substituted C_20_ since they have non-zero dipole moment and thus are expected to show appreciable changes in their spectra compared to those of C_20_ due to the large fraction of nitrogen. We perform anharmonic IR calculations for all species of the present study in their neutral, cationic, and anionic forms, both in the gas phase and in water solvent state. The calculated theoretical IR spectra are convolved with Lorentzian profiles of a full width at half maximum (FWHM) of 8 cm^−1^.^94^ We also estimate the electron affinity (E.A.) and ionization potential (I.P.) of these fullerenes, since these attributes are important parameters to control their chemical and physical properties.^95^ They are calculated by the following eqn (1) and (2).^96^

1 Ionization potential (I.P) = *E*_cation_ − *E*_neutral_

2 Electron affinity (E.A) = *E*_neutral_ − *E*_anion_

Here, *E*_neutral_, *E*_cation_, and *E*_anion_ represent the energies of optimized structures in their neutral, positively charged, and negatively charged forms, respectively. The electronic absorption spectra of these structures are reported using time-dependent density functional theory (TDDFT).^97^ The AOMix programme^98^ is used to identify the electronic transitions, oscillator strengths, and symmetry. The HOMO–LUMO energy gap is also obtained at the same level of theory.

## 3 Results and discussion

### 3.1 Molecular properties

**Fig. 1:**
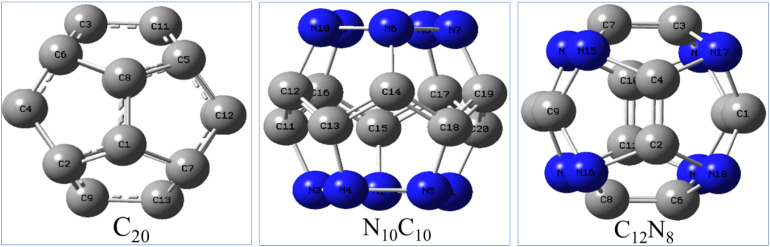
Structures of the C_20_ fullerene and its nitrogen-substituted heterofullerenes optimized at B3LYP/6-311++G(d,p) level of theory.

The molecular properties such as the energy, ionization potential, electron affinity and symmetry of C_20_ and its heterofullerenes in their neutral and ionic forms in gas and water solvent states are reported in Table 1, and the corresponding geometries are shown in Fig. 1. Unlike the highly stable C_60_, the C_20_ fullerene lacks hexagons and violates the isolated pentagon rule, making it less stable. Our calculations show that N-substituted forms (N_10_C_10_ and C_12_N_8_) exhibit enhanced stability, being consistent with previous reports.^31,69^ Nitrogen substitution improves electronic stability by acting as both electron donors and acceptors, which modifies the molecular orbital distribution. Among the heterofullerenes studied here, the C_12_N_8_ anion and the N_10_C_10_ cation exhibit notable dipole moments of 1.56 D and 0.35 D, respectively. In contrast, the neutral forms of both molecules show near-zero dipole moments, despite their low-symmetry geometries (*C*_1_ and *C*_2v_ point groups) in both gas and solvent phases. C_20_ fullerene exhibits the lowest symmetry *C*_i_ with zero dipole moment in neutral and ionic states. The fully optimized structure of the neutral C_12_N_8_ has *T*_h_ symmetry, while its anionic state has the lowest symmetry *C*_2v_.

**Table 1:** Energy relative to N_10_C_10_, ionization potential (I.P), electron affinity (E.A), and symmetry of the C_20_ fullerene and its N-heterofullerenes and their ions

Molecules	Neutral		Solvent		Cation		Anion		I.P (eV)	E. A (eV)
	Energy (eV)	Sym	Energy (eV)	Sym	Energy (eV)	Sym	Energy (eV)	Sym		
C_20_	4.526	*C*_i_	4.527	*C*_i_	4.525	*C*_i_	4.527	*C*_i_	6.95	2.32
N_10_C_10_	0.00	C_1_	0.00	C_1_	0.00	C_1_	0.00	C_1_	8.52	3.17
C_12_N_8_	9.02	*T*_h_	9.02	*T*_h_	9.01	*T*_h_	9.03	*C*_2v_	7.65	2.18

The average bond lengths of C_20_, C_12_N_8_, and N_10_C_10_ in their neutral, cationic, and anionic states (gas and solvent phases, harmonic and anharmonic levels) are summarized in Table S1. The pristine C_20_ cage shows uniform C–C bonds (∼1.45 Å), while nitrogen substitution introduces shorter C–N bonds (1.33–1.36 Å) and slightly perturbs C–C bonds (1.37–1.39 Å). In N_10_C_10_, N–N bonds appear (∼1.48 Å), further lowering the symmetry. Charge states influence bond lengths: in cations, C–C bonds contract by ∼0.01–0.02 Å, while in anions, bond alternation increases slightly. Solvent effects are minimal (<0.01 Å change). These results confirm that doping and charge localization significantly distort the C_20_ framework.

The results show that IR intensities are typically weak in the cationic states and significantly strong in the anionic forms, particularly for C–C stretching modes. Nitrogen substitution reduces molecular symmetry, leading to increased IR activity and changes the intensity patterns. The ionization potential (IP) of the nitrogen-substituted heterofullerenes N_10_C_10_ and C_12_N_8_ is higher than that of the C_20_ fullerene, amounting to 8.52 and 7.65 eV, respectively. It indicates that these molecules face strong resistance to losing electrons. In contrast, the neutral C_20_ fullerene exhibits a lower IP of 6.95 eV, indicating high nucleophilicity due to the concentration of positive charge at the centre of C_20_. The present calculation shows that C_20_ has an electron affinity of 2.32 eV, which agrees well with the experimental result of 2.25 eV.^50^ Miar *et al.*^99^ reported that nitrogen doping increases the electron affinity of the C_20_ fullerenes. In line with this, N_10_C_10_ exhibits a high electron affinity, indicating a strong tendency to accept electrons. Although N_10_C_10_ is overall neutral, its electron density is not evenly distributed. More electron density is concentrated near the center, which may affect how the molecule reacts. As a result, the carbon atoms show similar reactivity toward electrophiles.

The neutral C_20_, with its relatively low ionization potential (6.95 eV), can be described as nucleophilic, whereas nitrogen-substituted heterofullerenes (IP = 7.65–8.52 eV) are less nucleophilic and more electronically stable. The cationic species are electrophilic due to positive charge localization, while the anionic forms show strong nucleophilic character consistent with their higher electron density and electron affinities (Table 1).

### 3.2 Infrared spectra

**Fig. 2:**
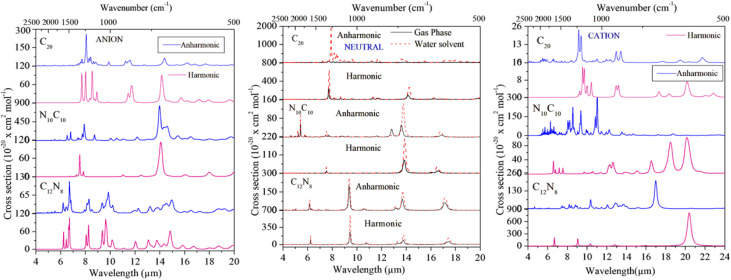
Infrared vibrational spectra of the C_20_ fullerene and its nitrogen-substituted heterofullerenes in their neutral gas and water solvent state, and ionic states. The spectra are calculated at the B3LYP/6-311++G(d,p) level of theory.

Harmonic and anharmonic mid-infrared absorption spectra for the neutral forms in both gas and water solvent phases, as well as harmonic IR spectra for the ionic states, are computed at the B3LYP/6-311++G(d,p) level of theory. These static DFT calculations are performed at 0 K, and the resulting spectra are presented in Fig. 2 over the 2–20 μm region. Anharmonic vibrational frequencies along with their relative intensities and combination modes for C_20_, N_10_C_10_, and C_12_N_8_ in the neutral and ionic forms are given in Table 2. Table 2 lists selected vibrational modes to highlight spectroscopically relevant features. Modes with near-zero IR intensity or degenerate components are omitted; the full set of 54 modes is provided in the SI. The vibrational analysis provides valuable insight into the structural characteristics of C_20_ and its nitrogen-substituted derivatives. Each molecule exhibits 54 vibrational modes, with several intense features highlighted in the main text. The complete set of harmonic vibrational frequencies and intensities in both gas and solvent phases is provided in the SI (Tables S3–S8, SI). These results are consistent with earlier studies.^28^

**Table 2:** The Anharmonic infrared vibrational frequencies (in cm^−1^) and relative intensity *I* (in kM mol^−1^) along with fundamental and combination modes for C_20,_ N_10_C_10,_ and C_12_N_8_ in their neutral form in the gas and water solvent states and their ionic forms

Neutral in gas phase			Neutral in water solvent			Cation in the gas phase			Anion in gas phase		
Frequency (cm^−1^)	Intensity (kM mol^−1^)	Mode^a^	Frequency (cm^−1^)	Intensity (kM mol^−1^)	Mode^a^	Frequency (cm^−1^)	Intensity (kM mol^−1^)	Mode^a^	Frequency (cm^−1^)	Intensity (kM mol^−1^)	Mode^a^
**C**_**20**_											
1310.49	49	V49	13 919.3	68	V51	1301.19	0.0001	V50	1360.98	0.0001	V54
		V1 + V49			V1 + V51			V19 + V50			—
1297.9	27	V50	1270.56	1811	V44	1265.97	0.5	V48	1299.1	47	V52
		V2 + V50			—			—			—
1289.65	29	V51	1237.39	173	V49	1265.11	0.6	V47	1254.22	30	V50
		V1 + V51			V1 + V49			V1 + V47			V2 + V50
1244.63	18	V44	1231.01	288	V38	1220.44	0.4	V44	1243.23	262	V49
		V1 + V44			V1 + V38			V22 + V44			V1 + V49
1240.26	29	V43	1208.54	434	V43	1203.04	9.1	V42	1191.12	62	V46
		V1 + V43			V1 + V43			V42 + V43			V2 + V46
1195.08	16	V42	1189	302	V50	1198.39	11	V41	1164.4	0.4	V39 + V48
		V2 + V42			V2 + V50			—			V1 + V39
		—			V2 + V36			—			—
1192.26	2	V39	1166.12	51	V35	1159.02	0.7	V38	1159.26	26	V41
		V2 + V39			V1 + V35			V22 + V38			—
1171.83	6	V38	1151.66	60	V42	1156.34	0.6	V39	1129.28	15	V38
		V1 + V38			V2 + V42			V22 + V39			V2 + V28
1159.21	3	V36	1117.83	6	V38	1104.53	0.001	V35	—	—	—
		V1 + V36			V1 + V38			V8 + V35			—
1078.04	1.6	V31	1044.6	167	V31	1037.47	1.5	V31	1117.49	1.2	V35
		V1 + V31			V1 + V31			—			V5 + V35
905.91	15	V28	924.19	23	V28			—			—
901.5	22	V27	858.29	218	V27	894.68	4	V28	893.87	33	V28
		—			V1 + V27			V28 + V52			V3 + V28
884.68	14	V26	898.83	87	V26	894	4	V27	877.68	27	V27
		—			V1 + V26			V2 + V27			V4 + V27
731.96	14	V21	740.84	150	V19 + V46	865.67	8	V26	866.3	44	V26
		V2 + V21			V1 + V19			V1 + V26			V5 + V26
726.54	12	V20	727.06	211	V20	679.45	0.3	V19	699.69	58	V21
		—			V1 + V20			V17 + V19			—
636.41	0.4	V2 + V18	657.07	82	V18	667.04	0.9	V18	685.34	0.5	V20
		—			V2 + V18			V18 + V40			V1 + V20
607.06	4	V14	646.55	27	V17	609.79	1.1	V14	622.73	18	V17
		V1 + V14			V1 + V17			V14 + V52			V17 + V37
582.15	7	V13	682.81	22	V13	608.58	0.1	V13	599.52	12	V14
		V2 + V13			V13 + V21			V13 + V46			V3 + V14
591.66	2	V12	584.32	153	V8	585.78	0.2	V12	572.49	0.5	V11
		V1 + V12			V2 + V8			V2 + V11			V2 + V11
		—			—			V12 + V43			V11 + V19
571.12	0.1	V10	573.45	41	V10 + V22	585.48	0.2	V11	572.48	0.4	V11 + V53
		V10 + V22			V1 + V10			V2 + V43			V11 + V19
569.2	5	V11	559.3	103	V9	567.16	0.007	V9	557.87	4	V12
		V1 + V11			V1 + V9			V9 + V17			V1 + V12
568.78	0.5	V9	551.65	14	V6	567.82	0.002	V10	542.35	6	V9
		V9 + V22			V1 + V6			V10 + V45			V9 + V18
557.56	0.2	V8	481.85	554	V11	564.41	0.002	V8	526.95	4	V8
		V1 + V8			V11 + V21			V8 + V35			V8 + V18
543.71	8	V6	464.21	32	V12	549.19	4	V7	510.38	9	V7
		V6 + V17			V1 + V12			—			—

**N**_**10**_ **C**_**10**_											
^d^1517.13	0.002	V1 + V54	^d^1524.76	0.001	V1 + V51	1775.93	14	V54	1533.44	50	V52
		V1 + V53			V1 + V53	1774.31	2	V53			V5 + V52
^d^1492.59	0.0002	V20 + V51	1495.79	0.4	V21 + V52	1737.57	47	V51	1474.29	199	V51
		V20 + V50									V1 + V51
1382.44	0.0013	V48	^d^1391.73	0.0002	V13 + V48	1642.53	165	V48	1357.05	50	V47
1346.6	2	V47			V22 + V47	1346.28	30	V41	1294.07	134	V46
1345.65	3	V46	1348.53	5.3	V46	1247.18	21	V36	1270.62	322	V53
1250.97	0.0002	V20 + V45	1257.6	0.009	V21 + V45	1205.57	331	V33	1261.02	128	V50
		—			—			V33 + V44			V5 + V50
984.91	0.0004	V2 + V43	1000.88	0.0001	V43	949.19	20	V27	1149.8	127	V48
		—			—			V27 + V44			V1 + V48
^d^925.87	0.1	V40	^d^929.06	1.1	V40	865.89	10.3	V24	948.12	8	V42
		V39			V39			V24 + V48			V41
859.31	5	V36	895.07	1.6	V36	860.46	3	V7 + V23	914	9	V37
733.75	26	V26	^d^726.59	72.2	V26	794.62	3.4	V17 + V20	832.22	16	V34
722.4	0.002	V29	^d^720.47	0.009	V29	723.49	6	V14	823.87	71	V35
^d^624.68	0.006	V19	^d^632.53	0.05	V19	618.8	1.1	V4 + V8	720.08	768	V26
592.18	9.3	V16	595.74	23	V16	537.19	2.6	V5	702.84	216	V2
485.94	0.3	V14 + V35	530.6	0.08	V5 + V14	518.65	5	V3 + V34	694.76	99	V25
		—			—			V3 + V27			—
347.34	0.1	V5	352.82	0.023	V5 + V14	—	—	—	688.03	245	V28
		—			—			—			V1 + V28
305.82	0.002	V2 + V43	308.6	0.6	V3 + V44	—	—	—	512.14	82	V14
		—			—			—	443.4	219	V7
		—			—			—	360.91	223	V5

**C**_**12**_ **N**_**8**_											
^d^1620.2	14	V50	^d^1619.15	23	V50	1353.49	22	V2 + V53	1618.55	13	V53
		V53			V51			—	1468.84	33	V50
		V52			V52			—			V6 + V54
1146.44	0.001	V4 + V47	1145.47	0.001	V4 + V47	1184.44	0	V47	1192.24	43	V4 + V47
1071.03	35	V42	1062.59	48	V41	1099.92	20.4	V40	1041.77	11	V7 + V42
^d^1069.4	42	V41	1061.96	56	V42	^d^1099.88	21	V41	1035.28	14	V8 + V37
		V40	1061.23	71	V40			V42	1009.14	5	V40
^d^938.51	3	V34	^d^938.55	5	V34	955.77	9.3	V35	913.27	0.3	V35
		V35			V35			V36			V2 + V35
		V36			V36	956.34	9	V34	909	1.1	V22 + V34
898.33	0	V23 + V31	899.13	0.1	V24 + V31	867.84	11	V1 + V31	819.29	15	V6 + V31
^d^759.07	6	V26	^d^761.2	11	V26	856.93	12	V3 + V29	763.93	9	V1 + V28
		V27			V27	853.58	14	V1 + V30			V28
^d^730.52	29	V23 + V31	^d^727.85	53	V23	759.75	2	V24 + V27	759.77	11	V3 + V26
		V24			V24 + V31	^d^752.41	1	V7 + V25	735.84	13	V24
		V25			V25 + V33			V23	725.42	14	V4 + V23
—	—	—	707.47	26	V2 + V15	^d^695.49	64	V27	706.49	8	V11 + V20
		—			—	694.67	66	V26	703.34	14	V21
		—			—	^d^684.82	1.7	V2 + V19	672.6	45	V17
^d^582.71	22	V11	582.82	36	V11	627.05	0.16	V11 + V15	537.22	12	V9 + V2
		V8 + V10			V7 + V10			V17 + V29			V9
		V7 + V9			V9			V7 + V25			—
^d^433.24	91	V4	^d^427.12	160	V4	^d^444.4	188	V9	406.05	166	V6
		V5			V5			V8	394.97	172	V4 + V5
		V6			V6			V7 + V25	305.98	70	V4 + V23

a Mode labels (*e.g.*, V49) follow the normal mode numbering from Gaussian output. Only a representative subset of vibrational modes is shown. Modes with negligible IR intensity or degenerate components are omitted for clarity. The complete set of 54 modes for each species is provided in the SI (Tables S3–S8).

Anharmonic effects lead to the appearance of combination bands and frequency shifts, as shown in Table 2. Unfortunately, experimental infrared spectra for the small fullerene C_20_ are not yet available. The following subsections present and discuss theoretically predicted anharmonic IR spectra of C_20_, N_10_C_10_, and C_12_N_8_ in their neutral, cationic, and anionic forms, both in the gas phase and in water solvent. We note that for some charged species, particularly the N_10_C_10_ anion, the HOMO–LUMO gap is relatively small (∼1.88 eV), which can raise concerns about low-lying excited states influencing the reliability of ground-state DFT vibrational spectra.^41^ However, all structures optimized in this study are confirmed to be true minima with no imaginary frequencies. The agreement of our calculated IR features with known vibrational bands in related carbonaceous molecules supports the validity of our results within the expected accuracy of the method. It is known that IR intensities often increase in fullerenes when they gain an electron (anion form), especially for C–C stretching modes. Our results follow this trend for C_20_ and its N-substituted forms. In contrast, PAH cations are known to show strong C–C stretches. In our case, nitrogen substitution changes the symmetry and causes the C–C stretch bands to become weaker or spread out, rather than becoming sharper.

#### 3.2.1 C_20_

Neutral C_20_ harmonic vibrational spectra have two intense IR-active modes: CC stretching at 1298 (7.7 μm) and 1296 (7.716 μm) cm^−1^, and CCC bending modes at 706 (14.16 μm) and 705 cm^−1^ (14.12 μm). The anharmonic infrared spectrum of neutral C_20_ in the water solvent phase shows that the most intense band appears at 1270.56 (7.87 μm) in 2–20 μm, which is attributed to a CC stretching mode of the V_41_ fundamental mode. The significantly intense mode in the spectra of C_20_ in water solvent is at 1319.3 cm^−1^ (7.58 μm), corresponding to the V_51_ fundamental and V_1_ + V_51_ combination modes of CC stretching. The ring distortion mode at 481.85 cm^−1^ (20.75 μm) with a large intensity corresponds to the fundamental mode V_11_ and a linear combination of the states V_11_ + V_21_.

#### 3.2.2 N_10_C_10_

The CN stretching mode has a strong intensity at 859.31 cm^−1^ (11.63 μm) and 733.75 cm^−1^ (13.62 μm), corresponding to the V_36_ and V_26_ fundamental modes, respectively. However, in the water solvent state, the fundamental mode V_26_ at 726.59 cm^−1^ (13.76 μm) has a quite intense peak. Other significant infrared features for this molecule in the cationic form are present in the CC stretching mode at 1642.53 cm^−1^ (6.09 μm) corresponding to the fundamental mode V_48_, and at 1205.57 cm^−1^ (8.279 μm) corresponding to the V_33_ + V_44_ combination and V_33_ fundamental modes. N_10_C_10_ in the anionic form shows significantly intense peaks at 1474.29 cm^−1^ (6.78 μm) and 1294.07 cm^−1^ (7.72 μm) corresponding to the V_51_ fundamental and V_1_ + V_51_ combinational modes, respectively. The ring distortion mode at 712 cm^−1^ becomes quite intense, which corresponds to the V_26_ fundamental mode.

#### 3.2.3 C_12_N_8_

The most intense IR bands for neutral C_12_N_8_ are present in the 2–20 μm region at 1620.2 cm^−1^ (6.17 μm), which is attributed to the V_50_ fundamental mode of CC stretching. The strong IR feature is dominated by the CN stretching modes at 1071.03 cm^−1^ (9.33 μm) and 1069.4 cm^−1^ (9.35 μm), corresponding to the V_41_ and V_42_ fundamental modes. The ring distortion feature appears at 433.24 cm^−1^ (23.08 μm). In the water solvent state, the CC stretching typically occurs at 1619.15 cm^−1^ (6.176 μm) corresponding to the V_50_, V_51_, V_52_ fundamental degenerated modes. The substitution of eight nitrogen atoms in the C_20_ fullerene introduces intense peaks with degeneracy at ∼1062 cm^−1^ (9.41 μm), corresponding to the V_40_, V_41_, and V_42_ fundamental modes. The ring distortion of C_12_N_8_ in water solvent is quite intense at 427.12 cm^−1^ (23.41 μm), compared to the neutral gas phase spectra.

### 3.3 Electronic absorption spectra

**Fig. 3:**
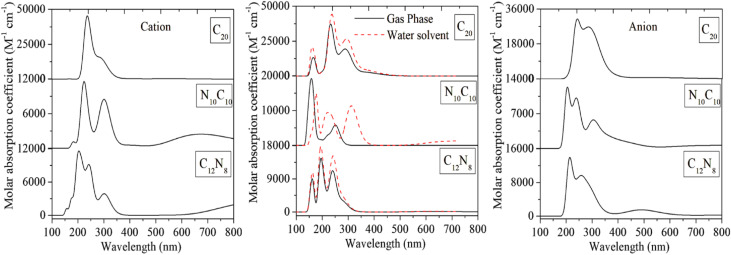
UV-Visible spectra of C_20_ fullerene and, its nitrogen-substituted heterofullerenes in their neutral and ionic states in the gas phase, and neutral in the water solvent. The spectra are calculated at the B3LYP/6-311++G(d,p) level of theory.

Electronic absorption spectra in the UV-visible region are studied using TD-DFT in the neutral and ionic states. The wavelength of the electronic transition, absorbance, oscillator strength, and the HOMO to LUMO gap of these molecules are summarized in Table 3, and corresponding spectra are shown in Fig. 3. The strong absorption bands from 150 to 300 nm are attributed to π → π* transitions.

**Table 3:** Wavelength of electronic transitions, absorbance, oscillator strength, and transitions of C_20_ fullerene and its nitrogen-substituted heterofullerenes

Molecule	Wavelength (nm)	Absorbance	Oscillator strength	Transitions	aH–L Energy gap (eV)
**Neutral in gas phase**					
C_20_	162.26	13 325	0.0746	H−1 → L+11	1.9168
	232.96	37 316	0.5118	H−3 → L+2	
	296.43	19 378	0.1882	H → L+7	
N_10_C_10_	158.83	19 306	0.0194	H−1 → L+15	4.5535
	255.16	5867	0.0409	H−1 → L+1	
C_12_N_8_	163.37	9115	0.0467	H−1 → L+17	2.8683
	195.46	14 662	0.0177	H−7 → L	
	241.35	11 257	0.0809	H−5 → L+1	

**Neutral in water solvent**					
C_20_	161.9	21 006	0.0673	H−1 → L+11	1.9252
	239.27	44 455	0.4408	H−3 → L+5	
	300.45	26 651	0.2749	H → L+7	
N_10_C_10_	177.32	15 156	0.1369	H → L+14	2.5565
	221.07	9446	0.1498	H−2 → L+4	
	310.8	11 382	0.1189	H−3 → L+1	
C_12_N_8_	164.18	10 855	0.0521	H−2 → L+17	2.9154
	192.05	17 931	0.0913	H−8 → L	
	242.47	15 284	0.1135	H−5 → L	

**Cation in gas phase**					
C_20_	236.14	45 400	0.3153	H−4 → L+5	1.8647
N_10_C_10_	180.72	1138	0.0181	H−7 → L+3	2.4153
	222.59	11 605	0.1042	H−2 → L+4	
	300.75	8484	0.0755	H−7 → L	
C_12_N_8_	157.58	1246	0.003	H−1 → L+13	2.3624
	215.27	11 624	0.0599	H−6 → L+5	
	244.12	9214	0.0504	H → L+7	
	302.8	3909	0.03	H−2→L+4	

**Anion in gas phase**					
C_20_	242.93	30 947	0.0503	H−5 → L+4	1.9282
	280.72	26 785	0.1073	H−2 → L+4	
N_10_C_10_	204.88	12 411	0.0727	H → L+14	1.8784
	236.03	10 191	0.0548	H−2 → L+4	
	307.95	5758	0.0569	H−8 → L	
C_12_N_8_	220.34	14 040	0.0162	H−6 → L+5	2.4
	263.38	9685	0.076	H → L+10	
	503.54	1517	0.0165	H → L+2	

a H and L represent HOMO and LUMO, respectively.

The C_20_ fullerene in the gas phase and its nitrogen substitution heterofullerenes N_10_C_10_ and C_12_N_8_ possess high-energy transitions at 162.26, 158.83, and 163.37 nm with major contributions from H−1 → L+11, H−1 → L+15, and H−1 → L+17, respectively. These high-energy transitions often result in significant changes in electronic configuration, indicating strong electronic coupling and potentially highly reactive excited states, which can affect the photostability, ionization, and dissociation behavior of these molecules in astrophysical environments where intense UV radiation is present.^100–102^ Due to nitrogen's high electronegativity, the energy gap between states increases, which influences electronic transitions in nitrogen-substituted heterofullerenes. There is no effect of water solvent on high-energy transitions of C_20_ and its heterofullerenes. Almost all transitions are observed in water solvent similarly to the gas phase state. In the case of ionic states especially in anion, there is no single high-energy transition observed. This may be because core-level electrons are unaffected by the addition of extra electrons. The C_20_ cation and its nitrogen heterofullerenes (N_10_C_10_ and C_12_N_8_) show high-energy transitions with major contributions from H−7 → L+3 and H−1 → L+13, respectively.

The HOMO–LUMO energy gap is a crucial parameter in determining the electronic properties of a molecule. A large HOMO–LUMO gap indicates higher kinetic stability with lower reactivity, representing greater chemical hardness.^103–105^ Compared to the parent fullerene C_20_, the N_10_C_10_ heterofullerene in the neutral gas phase shows a relatively large gap of 4.55 eV, indicating enhanced stability and reduced reactivity. In contrast, in the water solvent state N_10_C_10_ exhibits low kinetic stability, with a much smaller gap of 2.56 eV. Low-energy transitions are observed for all structures in the water solvent due to extended conjugation, electron delocalization, nitrogen substitution, and the absence of core-level transitions. In the case of cations and anions, almost all molecules exhibit lower energy transitions. Highly reactive and lowest stable species is N_10_C_10_ in anion, which shows the lowest HOMO–LUMO gap at 1.8784 eV, while the C_12_N_8_ heterofullerene has unique electronic properties, including a significantly large HOMO-to-LUMO energy gap in the anionic state due to a strong polarization between the N–C bonds. The position and number of substituted heteroatoms in the C_20_ fullerene affect the heterofullerenes HOMO–LUMO energy gap. This energy gap is also affected by the ionic charge states of the heterofullerene.

## 4 Astrophysical implications

The present results show that nitrogen-substituted C_20_ fullerenes in their anionic form exhibit strong IR-active modes, particularly in the 6–15 μm range. This suggests that such species may contribute to the observed interstellar infrared absorption features in cold or dense regions, where anionic forms are more stable due to the shielding from UV radiation; however, their role in emission spectra is likely limited to environments with large UV excitation. The enhanced IR activity, resulting from the symmetry reduction *via* nitrogen substitution, increases their detectability compared to the pure C_20_. Furthermore, our calculated ionization potentials (IP) and electron affinities (EA) support the idea that anionic species are favored in shielded environments, while cationic forms are more likely to exist in UV-irradiated regions such as photodissociation regions. These findings suggest the variations in charge states and IR features of these molecules across different astrophysical environments. The detection of fullerenes C_60_ and C_70_ in the planetary nebula TC-1 by Cami *et al.*^6^ marked a major step forward in understanding fullerene chemistry in space. Although indene was recently detected in the TMC-1 molecular cloud,^106^ no gas-phase formation pathway for fullerene-like molecules is currently known under such low-temperature conditions. In support of this, Lorenzo *et al.*^107^ experimentally demonstrated the formation of the C_20_ fullerenes and small carbon clusters in laser-induced plasma, mimicking the conditions in the vicinity of the central star of planetary nebulae. These developments suggest that small fullerenes like C_20_ and its derivatives are likely to be present in space, and the present anharmonic calculations provide important spectral fingerprints to aid their future identification.

In dense molecular clouds, dust grains are covered with ice mantles primarily composed of H_2_O, CO, and CO_2_.^108–111^ Numerous observations using ISO-SWS and Spitzer confirm that H_2_O, CO, and CO_2_ are the most abundant and ubiquitous molecules frozen in mantles on interstellar grains.^112,113^ After absorbing cosmic rays or UV radiation, the surface reactions of frozen molecules could lead to the formation of carbonaceous material containing fullerenes.^110^ We observed that neutral anharmonic C_20_ fullerene has strong features at around 7.7, 8.0, 8.6, 9.2, and 11.3 μm in the gas and around 7.8, 8.0, 8.6, 9.6, and 10.8 μm in the water solvent state. Although the water solvent (ice mantle) has an insignificant effect on the peak positions of C_20_, this implies that distinguishing its presence in icy *versus* gas-phase environments solely based on vibrational band positions will be challenging, especially in absorption studies where precise shifts are crucial. The features of the cationic form appear at around 7.8, 8.2, 8.6, 11.2, and 17.1 μm with very weak intensity, while the anionic form exhibits features with significant intensity at 7.7, 8.03, 8.6, 11.2, and 18.9 μm. The vibrational spectra of the heterofullerene N_10_C_10_ display intense peaks at ∼6.1, ∼8.2, 10.5, and 12.6 μm in the cationic form. In the anion state, it shows strong features at 7.8, 8.6, and 10.5 μm. While the neutral form of C_12_N_8_ in the water solvent state shows strong features at around 6.2 and 10.5 μm, weak features are also observed at ∼8.7 and ∼11.2 μm. On the other hand, CN and CC stretching vibrational modes are quite intense, peaking at 6.8, 8.4 μm, and 14.86, 18.61 μm in the C_12_N_8_ anionic form. The differences in intensities among the vibrational spectra of nitrogen-containing molecules could be attributed to the position and number of heteroatoms in the parent fullerene C_20_. Experimental and theoretical studies by Mattioda *et al.*, Hudgins *et al.*, and Vats *et al.*^114–116^ suggest that nitrogen-containing PAHs (PANHs), in neutral, cationic, and anionic forms, may contribute to the aromatic infrared bands (AIBs) observed in the interstellar medium (ISM). Recent JWST observations of the Orion Bar have revealed high-resolution AIB spectra that help constrain potential carriers more precisely.^117^ Furthermore, carbon-rich dust containing nitrogen, with infrared features similar to those seen in novae, underscores the astrophysical importance of nitrogenated species.^118^

**Fig. 4:**
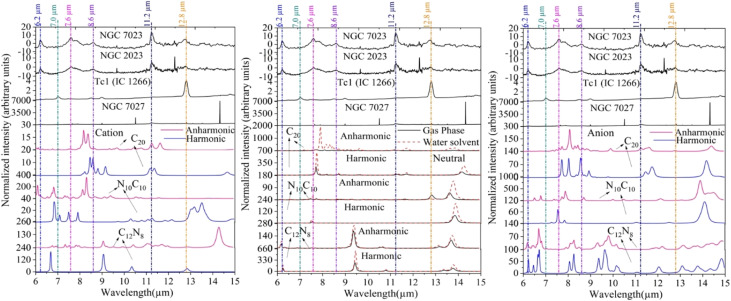
Theoretical vibrational spectra of the C_20_ fullerene and its nitrogen-substituted heterofullerenes in their neutral (gas phase and water solvent) and ionic states, compared with the observed spectra of four astronomical objects. The vertical dotted lines mark the positions of the interstellar aromatic infrared bands (AIBs), including the 7.0 μm band attributed to neutral C_60_. Observational spectra are plotted in surface brightness units (MJy sr^−1^), while theoretical spectra are shown as normalized intensities (a.u.) to facilitate direct comparison.

To explore the potential presence of C_20_ and its nitrogen-substituted derivatives in space, we selected two planetary nebulae (Tc 1 and NGC 7027) and two reflection nebulae (NGC 2023 and NGC 7023), based on their well-characterized mid-infrared emission features. These objects are ideal for comparison with the computed spectra of C_20_ fullerenes in neutral and ionic states. A summary of their key physical properties and observed IR bands is provided in Table 4. Their spectra are compared with the theoretical results in Fig. 4 to evaluate possible spectral matches. While some agreement is seen, further observational confirmation is required. A direct comparison of the computed spectra with the observed IR spectra of these nebulae is presented in Fig. 4. This comparison suggests that C_20_ and its N-substituted species could contribute to some of the observed features in these astronomical sources. However, due to the current computational limitations, the assignments remain tentative. Additional experimental and observational efforts are needed—particularly using high-resolution and high-signal-to-noise-ratio spectra-to confirm the presence of these species and refine their spectroscopic identification.

**Table 4:** Characteristics of the objects studied

Object	Object	Type	Teff (K)	Detected features (μm)					
TC1	Planetary nebula	34 700 (ref. 123)	6.23	7.0 (ref. 6)	7.7	8.51	8.6	11.3	
NGC 7027	Planetary nebula	200 000 (ref. 124 and 125)	6.2	—	7.65 (ref. 126)	—	8.6	11.3	12.6
NGC 2023	Reflection nebula	22 000 (ref. 5)	6.2	—	7.6	—	8.6	11.25	
NGC 7023	Reflection nebula	17 000 (ref. 127)	6.2	—	7.7	—	8.6	11.25	

The UV bump at 217.5 nm in the interstellar extinction curve is attributed to the carbonaceous dust grains in space, although exact identification of the carrier remains an open question.^63^ Massa *et al.*^119^ show that the area of the 217.5 nm extinction bump and the strengths of the major AIB arise show a strong correlation for the same lines of sight, suggesting common carriers for both the UV extinction and the AIB emission. However, observational searches for PAH signatures in 400–700 nm have so far been unsuccessful in the interstellar extinction.^66,120–122^ Theoretical UV-visible spectra for the C_20_ fullerene and heterofullerenes show that the neutral C_20_ appears to have significant absorption at 232.96 nm, whereas in the water solvent, it is at 221.07 nm for N_10_C_10_. Apart from this, in the ionic state, the UV bump is observed at 222.59 nm for N_10_C_10_ and 215.27 nm for C_12_N_8_ in the cationic state, while in the anionic form, it is at 220.34 nm for C_12_N_8_. These results suggest that part of the 217.5 nm may have a contribution from C_20_ fullerene and heterofullerenes. Non-detection of the second strong feature around 300 nm suggests that the contribution should be limited. Further studies of the C_20_ fullerene, particularly in laboratory experiments, are needed to make a detailed study of the presence of the C_20_ fullerene in the ISM. The present study provides UV to IR spectra of the C_20_ fullerene and heterofullerenes for future studies of the possible presence of these small carbon clusters. These species may be able to survive in the presence of other fullerenes in the interstellar medium and circumstellar envelopes.

## 5 Summary

The discovery of C_60_, C_60_^+^, and C_70_ fullerenes in interstellar and circumstellar environments opened a new window to study the formation and evolution of carbonaceous species in the ISM. It suggests possibilities for the presence of other fullerenes and their derivatives. We conduct a theoretical study on the C_20_ fullerene and its nitrogen-substituted heterofullerenes for a comprehensive analysis of IR vibrational and electronic absorption spectral features. The analyses are performed for both the neutral and ionic states in the gas state, and also for the neutral water solvent state using the B3LYP/6-311++G(d,p) level of theory taking account of the anharmonic effects. We find significant effects in the vibrational and electronic absorption spectral features, when nitrogen-substitution occurs in the parent fullerene C_20_. The present results also show that C_12_N_8_ has a significant dipole moment in the anionic state with the lowest symmetry. The electron affinity of C_20_ is 2.32 eV, which is very close to the experimental measurement.

We compare the IR spectra observed in four astronomical objects with those of the C_20_ fullerene and heterofullerenes obtained in this study for the neutral and charge states. The wavelengths of strong vibrational modes for three molecules in their neutral and ionic states have peaks close to the peaks at 6.2, 6.6, 7.0, 7.7, 8.5, 8.6, 11.2, and 11.3 μm observed in astronomical objects. IR spectroscopic observations with the James Webb Space Telescope (JWST) will constitute a significant advancement in observational astronomy owing to their exceptional sensitivity and resolution. A vast array of astrophysical domains will be significantly impacted by the capacity to identify certain species, estimate their abundances or provide upper bounds on non-detections. This epoch holds the potential to augment our comprehension of the chemical composition of the universe, the mechanisms behind the development of stars and planets, the progression of galaxies, and the underlying essence of the cosmos.

We also report the electronic absorption spectra of these isomers using the TDDFT for all isomers in neutral and their ionic charge states. The heterofullerenes in the neutral form show 221.07 nm for N_10_C_10_ in the water solvent. In ionic states, these molecules have a broad absorption bump at 222.59 nm for N_10_C_10_ and 215.27 nm for C_12_N_8_ in cationic form, and in the anionic form at 220.34 nm for C_12_N_8_. The substitution of many heteroatoms in the C_20_ fullerene significantly affects the HOMO–LUMO energy gap in the gas phase, and this energy gap is also strongly affected by the water solvent and ionic charge states of the molecules. The HOMO-to-LUMO energy gap of a neutral N_10_C_10_ exhibits a high energy gap of 4.5535 eV in the gas phase, but it is observed in the water solvent as 1.9252 eV. Changes in the energy gap can influence the molecule's chemical stability and its ability to participate in electronic transitions. Larger gaps often correspond to more stable structures, while smaller gaps may enhance reactivity, which is important for catalysis or chemical sensing.

## Conflicts of interest

The authors declare that they have no known competing financial interests or personal relationships that could have appeared to influence the work reported in this paper.

## Supplementary Material

RA-015-D5RA05271H-s001

## Data Availability

The data that support the findings of this study are available within the article and its supplementary information (SI) files. Additional data, including computational input/output files, optimized geometries, and detailed numerical results, are available from the corresponding author upon reasonable request. Supplementary information is available. See DOI: https://doi.org/10.1039/d5ra05271h.
