# Supplementary material for: C20 and nitrogen-substituted fullerenes: anharmonic IR and UV-vis spectra for astrophysical environments
Source: RSC Adv. 2025 Oct 28;15(48):41156–68. doi: 10.1039/d5ra05271h (PMC12560090; doi:10.1039/d5ra05271h)
Supplement: RA-015-D5RA05271H-s001 [file RA-015-D5RA05271H-s001.pdf]

Supplementary Information (SI) for RSC Advances.
This journal is © The Royal Society of Chemistry 2025

Supplementary Information

C_{20} and Nitrogen-Substituted Fullerenes: Anharmonic IR and UV–Vis Spectra for Astrophysical Environments

Venkata Lakshmi Karri[1], Ajay Chaudhari[2], Takashi Onaka[3], Mahadevappa Naganathappa[1]*

Table S1. Average bond length (in Å) of CC for C_{20} fullerene, CC & CN for $C_{12}N_8$, and CC, CN, &NN for $N_{10}C_{10}$ fullerene

States	Type	C_{20}	$C_{12}N_8$		$N_{10}C_{10}$		
		CC	CC	CN	CC	CN	NN
Neutral In Gas Phase	Harmonic	1.451	1.329	1.481	1.374	1.486	1.49
	Anharmonic	1.451	1.329	1.481	1.374	1.486	1.49
Neutral In Solvent	Harmonic	1.451	1.329	1.481	1.373	1.485	1.486
	Anharmonic	1.451	1.329	1.481	1.373	1.485	1.486
Cation In Gas Phase	Harmonic	1.447	1.34	1.46	1.379	1.477	1.48
	Anharmonic	1.456	1.34	1.46	1.379	1.477	1.48
Anion In Gas Phase	Harmonic	1.456	1.355	1.459	1.387	1.477	1.48
	Anharmonic	1.456	1.355	1.459	1.387	1.477	1.48

Table S2. Cartesian coordinates (in Å) for C_{20} fullerene and its nitrogen-substituted heterofullerenes $N_{10}C_{10}$ and $C_{12}N_8$ molecules optimized at B3LYP/6-311++G (d, p) level of theory in the neutral and ionic charge states in the gas phase, and water solvent states.

C_{20}	Neutral			Solvent		
Carti Coord	X	Y	Z	X	Y	Z
C_1	1.186582	-0.412221	1.658415	1.186289	-0.411394	1.657246
C_2	0.057343	-1.24307	1.658614	0.056214	-1.242764	1.657941
C_3	-1.179827	1.671499	0.377787	-1.179468	1.670361	0.37799
C_4	-1.094351	-0.363424	1.606323	-1.095777	-0.364261	1.606604
C_5	1.101064	1.596313	0.386988	1.102578	1.597187	0.386577
C_6	-0.696073	1.025371	1.662972	-0.696064	1.024835	1.662294
C_7	1.838308	-0.629282	0.382983	1.839007	-0.628266	0.382463
C_8	0.70722	1.0276	1.656612	0.707635	1.027775	1.656211
C_9	0.053075	-2.039267	0.370972	0.051961	-2.038936	0.370904
C_{10}	-1.808087	-0.597073	0.375348	-1.809549	-0.597957	0.375747
C_{11}	-0.053075	2.039267	-0.370972	-0.051961	2.038936	-0.370904
C_{12}	1.808087	0.597073	-0.375348	1.809549	0.597957	-0.375747
C_{13}	1.179827	-1.671499	-0.377787	1.179468	-1.670361	-0.37799
C_{14}	-1.101064	-1.596313	-0.386988	-1.102578	-1.597187	-0.386577
C_{15}	-1.838308	0.629282	-0.382983	-1.839007	0.628266	-0.382463
C_{16}	-0.057343	1.24307	-1.658614	-0.056214	1.242764	-1.657941
C_{17}	1.094351	0.363424	-1.606323	1.095777	0.364261	-1.606604
C_{18}	0.696073	-1.025371	-1.662972	0.696064	-1.024835	-1.662294
C_{19}	-0.70722	-1.0276	-1.656612	-0.707635	-1.027775	-1.656211
C_{20}	-1.186582	0.412221	-1.658415	-1.186289	0.411394	-1.657246
	Cation			Anion		
C_1	1.179409	-0.382763	1.591035	-0.305303	1.634339	1.124237
C_2	0.026327	-1.229438	1.598804	-1.612169	1.087118	0.699604
C_3	-1.238527	1.690717	0.407609	0.406592	0.590872	-1.814588
C_4	-1.158612	-0.373951	1.607563	-1.613623	1.085608	-0.698747
C_5	1.149162	1.614286	0.374763	1.67966	1.145464	-0.002766
C_6	-0.723617	1.000494	1.59137	-0.307728	1.631713	-1.127028
C_7	1.882647	-0.620882	0.355081	0.410481	0.594911	1.812659

C_8	0.718086	1.00415	1.594941	0.470216	2.06735	-0.002556
C_9	0.031984	-1.979813	0.367426	-1.699555	-0.329342	1.177694
C_{10}	-1.88044	-0.610427	0.39512	-1.702152	-0.332052	-1.173761
C_{11}	-0.031984	1.979813	-0.367426	1.699555	0.329342	-1.177694
C_{12}	1.88044	0.610427	-0.39512	1.702152	0.332052	1.173761
C_{13}	1.238527	-1.690717	-0.407609	-0.406592	-0.590872	1.814588
C_{14}	-1.149162	-1.614286	-0.374763	-1.67966	-1.145464	0.002766
C_{15}	-1.882647	0.620882	-0.355081	-0.410481	-0.594911	-1.812659
C_{16}	-0.026327	1.229438	-1.598804	1.612169	-1.087118	-0.699604
C_{17}	1.158612	0.373951	-1.607563	1.613623	-1.085608	0.698747
C_{18}	0.723617	-1.000494	-1.59137	0.307728	-1.631713	1.127028
C_{19}	-0.718086	-1.00415	-1.594941	-0.470216	-2.06735	0.002556
C_{20}	-1.179409	0.382763	-1.591035	0.305303	-1.634339	-1.124237

$N_{10}C_{10}$	Neutral			Solvent		
Carti Coord	X	Y	Z	X	Y	Z
N_1	-0.78383569	-1.85100019	-1.16365056	1.747647	1.31924	0.073135
N_2	-1.6248049	-1.5467499	0.12649071	1.747966	0.477287	-1.231577
N_3	-0.72015668	-1.67515908	1.38237274	1.748082	-1.024404	-0.83372
N_4	0.73242378	-2.00160389	0.87705542	1.748137	-1.109888	0.716214
N_5	0.69188952	-2.0202955	-0.6943519	1.747505	0.338867	1.276827
N_6	1.60924985	1.5393441	-0.30323253	-1.747755	-0.477283	1.231764
N_7	0.54205951	1.74461171	-1.42486843	-1.748267	1.024173	0.834145
N_8	-0.83222106	1.96081911	-0.72339878	-1.748132	1.109635	-0.716813
N_9	-0.5764095	2.07417186	0.8296916	-1.747371	-0.33847	-1.277007
N_{10}	0.95123462	1.77699777	1.08171448	-1.747817	-1.319076	-0.07298
C_{11}	-0.43719481	-0.2019584	1.52023727	0.369424	-1.453062	-1.182895
C_{12}	0.7109519	0.32314768	1.38840311	-0.368989	-1.871845	-0.1033
C_{13}	1.19347108	-0.57042461	0.86239102	0.369164	-1.574479	1.016099
C_{14}	1.51238476	0.07558352	-0.19662502	-0.369056	-0.676372	1.747278
C_{15}	-1.5147515	-0.08047776	0.10429626	0.369346	0.676248	-1.747016
C_{16}	-1.01585104	0.64771518	1.02388805	-0.368734	-0.480042	-1.810927
C_{17}	-1.20954294	0.5426666	-0.75352914	-0.369154	1.574603	-1.016336
C_{18}	1.05629735	-0.61498441	-0.93930719	0.368903	0.480065	1.810664
C_{19}	0.34499698	0.23818552	-1.56318697	-0.369605	1.453157	1.183136
C_{20}	-0.63019124	-0.36058931	-1.43432015	0.368705	1.871633	0.103312

	Cation			Anion		
N_1	1.763183	1.211887	-0.299584	1.670877	-0.678444	-1.224583
N_2	1.871985	-0.078704	-1.143755	1.596689	-1.457014	0.121801
N_3	1.74377	-1.255633	-0.155979	1.710227	-0.436779	1.292556
N_4	1.584798	-0.685479	1.225976	1.855414	1.008757	0.673342
N_5	1.594513	0.805102	1.140517	1.829942	0.8529	-0.902962
N_6	-1.88592	0.0794	1.154582	-1.597166	1.456916	-0.119095
N_7	-1.755362	1.258312	0.151553	-1.71046	0.433947	-1.292991
N_8	-1.563434	0.664864	-1.198078	-1.854879	-1.007957	-0.675683
N_9	-1.575712	-0.782867	-1.117752	-1.830693	-0.851902	0.902746
N_{10}	-1.776174	-1.216793	0.29677	-1.669973	0.679678	1.224817
C_{11}	0.414067	-1.801169	-0.428523	0.339071	-0.430468	1.840974
C_{12}	-0.440109	-1.785476	0.647708	-0.282596	0.773217	1.718404
C_{13}	0.202327	-0.994084	1.580677	0.546084	1.631933	0.974787
C_{14}	-0.566261	0.1127	1.866623	-0.182197	1.86221	-0.179241
C_{15}	0.563338	-0.110795	-1.837877	0.181432	-1.862337	0.179663
C_{16}	-0.213582	-1.203281	-1.508893	-0.510315	-1.4063	1.278239
C_{17}	-0.198929	1.023414	-1.637631	-0.545183	-1.632288	-0.975403
C_{18}	0.216358	1.168177	1.455126	0.508977	1.406985	-1.278452
C_{19}	-0.416531	1.853044	0.434968	-0.339206	0.429953	-1.840937
C_{20}	0.437401	1.737367	-0.635469	0.28396	-0.773021	-1.717975

$C_{12}N_8$	Neutral			Solvent		
Carti Coord	X	Y	Z	X	Y	Z
C_1	0	0.664768	-1.799819	0	0.664768	-1.799819
C_2	0.664768	1.799819	0	0.664768	1.799819	0
C_3	-1.799819	0	-0.664768	-1.799819	0	-0.664768
C_4	-0.664768	1.799819	0	-0.664768	1.799819	0
C_5	0	-0.664768	-1.799819	0	-0.664768	-1.799819
C_6	1.799819	0	-0.664768	1.799819	0	-0.664768
C_7	-1.799819	0	0.664768	-1.799819	0	0.664768
C_8	1.799819	0	0.664768	1.799819	0	0.664768
C_9	0	0.664768	1.799819	0	0.664768	1.799819
C_{10}	-0.664768	-1.799819	0	-0.664768	-1.799819	0
C_{11}	0.664768	-1.799819	0	0.664768	-1.799819	0
C_{12}	0	-0.664768	1.799819	0	-0.664768	1.799819
N_1	-1.245593	-1.245593	1.245593	-1.245593	-1.245593	1.245593
N_2	1.245593	-1.245593	1.245593	1.245593	-1.245593	1.245593
N_3	-1.245593	1.245593	1.245593	-1.245593	1.245593	1.245593
N_4	1.245593	1.245593	1.245593	1.245593	1.245593	1.245593
N_5	-1.245593	1.245593	-1.245593	-1.245593	1.245593	-1.245593
N_6	1.245593	1.245593	-1.245593	1.245593	1.245593	-1.245593
N_7	-1.245593	-1.245593	-1.245593	-1.245593	-1.245593	-1.245593
N_8	1.245593	-1.245593	-1.245593	1.245593	-1.245593	-1.245593
		Cation			Anion	
C_1	0	0.670387	-1.812607	0	-1.720992	-0.625054
C_2	0.670387	1.812607	0	0.717625	0	-2.011409
C_3	-1.812607	0	-0.670387	-1.784887	-0.664785	-0.00599
C_4	-0.670387	1.812607	0	-0.717625	0	-2.011409
C_5	0	-0.670387	-1.812607	0	-1.891152	0.713536
C_6	1.812607	0	-0.670387	1.784887	-0.664785	-0.00599
C_7	-1.812607	0	0.670387	-1.784887	0.664785	-0.00599
C_8	1.812607	0	0.670387	1.784887	0.664785	-0.00599
C_9	0	0.670387	1.812607	0	1.720992	-0.625054
C_{10}	-0.670387	-1.812607	0	-0.670066	0	1.803686
C_{11}	0.670387	-1.812607	0	0.670066	0	1.803686
C_{12}	0	-0.670387	1.812607	0	1.891152	0.713536
N_1	-1.216912	-1.216912	1.216912	-1.261662	1.236603	1.266032
N_2	1.216912	-1.216912	1.216912	1.261662	1.236603	1.266032
N_3	-1.216912	1.216912	1.216912	-1.196438	1.23821	-1.209795
N_4	1.216912	1.216912	1.216912	1.196438	1.23821	-1.209795
N_5	-1.216912	1.216912	-1.216912	-1.196438	-1.23821	-1.209795
N_6	1.216912	1.216912	-1.216912	1.196438	-1.23821	-1.209795
N_7	-1.216912	-1.216912	-1.216912	-1.261662	-1.236603	1.266032
N_8	1.216912	-1.216912	-1.216912	1.261662	-1.236603	1.266032

Each vibrational mode is labeled as fundamental (F) or combination band (CB). All 54 modes for each molecule (fundamentals and combination bands) are listed here; missing labels in earlier versions have been corrected.

Table S3. The harmonic and anharmonic infrared vibrational frequencies (in cm^{-1}) with relative intensity I (in KM mol^{-1}), including fundamental and combination modes for neutral C_{20} fullerene in the gas and water solvent states.

C_{20}	Neutral in the gas phase			Neutral in water solvent		
Vibrational Modes	Harmonic Freq (Int)	Anharmonic Freq (Int)	Mode	Harmonic Freq (Int)	Anharmonic Freq (Int)	Mode
C-C stretch	1376(0)	1378(0)	V_{54} (F)	1375(0)	1445(0)	V_{48} (F)
			V_6+V_{54} (CB)			V_9+V_{48} (CB)
	1374(0)	1372(0)	V_{53} (F)	1373(0)	1400(0)	V_{54} (F)
			V_6+V_{53} (CB)			$V_{18}+V_{54}$ (CB)
	1350(0)	1343(0)	V_{52} (F)	1349(0)	1319(68)	V_{51} (F)
			V_6+V_{52} (CB)			V_1+V_{51} (CB)
	1298(118)	1310(48)	V_{49} (F)	1292(346)	1288(0)	V_{46} (F)
			V_1+V_{49} (CB)			$V_{19}+V_{46}$ (CB)
			V_2+V_{49} (CB)			$V_{20}+V_{46}$ (CB)
	1296(122)	1298(27)	V_{50} (F)	1289(343)	1277(0)	V_{53} (F)
			V_2+V_{50} (CB)			$V_{18}+V_{53}$ (CB)
	1289(4)	1289(29)	V_{51} (F)	1284(5)	1270(1811)	V_{44} (F)
			V_1+V_{51} (CB)			V_1+V_{44} (CB)
	1255(0)	1283(0)	V_{48} (F)	1250(0)	1244(0)	V_{41} (F)
			$V_{48}+V_{50}$ (CB)			$V_{20}+V_{41}$ (CB)
	1249(0)	1276(0)	V_{45} (F)	1246(0)	1237(173)	V_{49} (F)
			$V_{21}+V_{45}$ (CB)			V_1+V_{49} (CB)
	1236(0)	1244(18)	V_{44} (F)	1234(0)	1231(288)	V_{38} (F)
			V_1+V_{44} (CB)			V_1+V_{38} (CB)
	1229(0)	1240(30)	V_{43} (F)	1227(0)	1214(0)	V_{52} (F)
			V_1+V_{43} (CB)			V_6+V_{52} (CB)
	1228(3)	1223(0)	V_{41} (F)	1226(11)	1208(434)	V_{43} (F)
			$V_{20}+V_{41}$ (CB)			$V_{19}+V_{47}$ (CB)
			---			V_2+V_{43} (CB)
	1226(6)	1195(16)	V_{42} (F)	1225(20)	1189.6(0)	V_{47} (F)
			V_2+V_{42} (CB)			V_2+V_{50} (CB)
	1199(2)	1192(2)	V_{39} (F)	1199(5)	1189(301)	V_{50} (F)
			V_1+V_{39} (CB)			V_2+V_{50} (CB)
			V_2+V_{39} (CB)			---

	1173(0)	1191(0)	V_{46} (F)	1173(0)	1179(6)	V_{36} (F)
			$V_{19}+V_{46}$ (CB)			$V_{1}+V_{36}$ (CB)
			$V_{20}+V_{46}$ (CB)			$V_{2}+V_{36}$ (CB)
	1171(0)	1185(0)	V_{18} (F)	1171(0)	1166(50)	V_{35} (F)
			$V_{18}+V_{40}$ (CB)			$V_{1}+V_{35}$ (CB)
			---			$V_{2}+V_{35}$ (CB)
	1151(10)	1181(0)	V_{47} (F)	1150(29)	1162(0)	V_{37} (F)
			$V_{10}+V_{47}$ (CB)			$V_{6}+V_{37}$ (CB)
	1150(9)	1171(6)	V_{38} (F)	1149(26)	1151(60)	V_{42} (F)
			$V_{1}+V_{38}$ (CB)			$V_{2}+V_{42}$ (CB)
			$V_{21}+V_{47}$ (CB)			---
	1126(0)	1159(3)	V_{36} (F)	1126(0)	1117(95)	V_{39} (F)
			$V_{1}+V_{36}$ (CB)			$V_{1}+V_{39}$ (CB)
			$V_{2}+V_{36}$ (CB)			$V_{2}+V_{39}$ (CB)
CCC bend	1118(0.2)	1150(0)	V_{37} (F)	1118(0.2)	1097.2(48)	V_{34} (F)
			$V_{6}+V_{37}$ (CB)			$V_{1}+V_{34}$ (CB)
			---			$V_{3}+V_{34}$ (CB)
	1113(2)	1124(0.6)	V_{35} (F)	1113(5)	1097(0)	V_{30} (F)
			$V_{2}+V_{35}$ (CB)			$V_{6}+V_{30}$ (CB)
	1112(2)	1102(0.4)	V_{34} (F)	1112(6)	1080(0)	V_{40} (F)
			$V_{2}+V_{34}$ (CB)			$V_{18}+V_{40}$ (CB)
	1079(0)	1078(1.5)	V_{31} (F)	1079(0)	1044(166)	V_{31} (F)
			$V_{1}+V_{31}$ (CB)			$V_{1}+V_{31}$ (CB)
	1078(0)	1076(0)	V_{32} (F)	1078(0)	1004(0)	V_{32} (F)
			$V_{31}+V_{32}$ (CB)			$V_{19}+V_{32}$ (CB)
	1040(7)	1073(0)	V_{33} (F)	1040(20)	986(0)	V_{33} (F)
			$V_{31}+V_{33}$ (CB)			$V_{31}+V_{33}$ (CB)
	963(0)	1001(0)	V_{30} (F)	958(0)	924(23)	V_{28} (F)
			$V_{6}+V_{30}$ (CB)			$V_{2}+V_{28}$ (CB)
	959(0)	993(0)	V_{29} (F)	955(0)	902(0)	V_{29} (F)
			$V_{6}+V_{29}$ (CB)			$V_{6}+V_{29}$ (CB)
	887(8)	905(15)	V_{28} (F)	886(24)	899(87)	V_{26} (F)
			$V_{28}+V_{52}$ (CB)			$V_{1}+V_{26}$ (CB)
	885(9)	901(22)	V_{27} (F)	885(27)	858(218)	V_{27} (F)
			$V_{1}+V_{27}$ (CB)			$V_{1}+V_{27}$ (CB)

854(25)	884(14)	V_{26} (F)	853(70)	823(87)	V_{25} (F)
		V_2+V_{26} (CB)			$V_{17}+V_{25}$ (CB)
796(0)	820(0)	V_{25} (F)	796(0)	784(0)	V_{24} (F)
		$V_{16}+V_{25}$ (CB)			V_9+V_{24} (CB)
755(0)	774(0)	V_{24} (F)	755(0)	761(0)	V_{23} (F)
		V_9+V_{24} (CB)			V_9+V_{23} (CB)
754(0)	770(0)	V_{23} (F)	754(0)	752(0)	V_{45} (F)
		V_9+V_{23} (CB)			$V_{31}+V_{45}$ (CB)
743(0)	734(0)	V_{19} (F)	745(0)	740(150)	V_{19} (F)
		$V_{11}+V_{19}$ (CB)			V_1+V_{19} (CB)
706(45)	732(13)	V_{21} (F)	704(0)	736(0)	V_{21} (F)
		V_2+V_{21} (CB)			$V_{12}+V_{21}$ (CB)
705(42)	726(12)	V_{20} (F)	703(134)	727(210)	V_{20} (F)
		V_2+V_{20} (CB)			V_1+V_{20} (CB)
703(0)	720(14)	V_{22} (F)	702(129)	710(0)	V_6+V_{15} (CB)
		$V_{10}+V_{22}$ (CB)			$V_{16}+V_{15}$ (CB)
629(1.3)	665(0)	V_{17} (F)	629(3)	704(150)	V_{46} (F)
		V_6+V_{17} (CB)			$V_{19}+V_{46}$ (CB)
		$V_{17}+V_{43}$ (CB)			---
616(0)	636(0.4)	V_{18} (F)	615(16)	682(21)	V_{13} (F)
		V_2+V_{18} (CB)			V_1+V_{13} (CB)
		$V_{18}+V_{40}$ (CB)			---
615(5)	627(0)	V_{16} (F)	612(17)	668(15)	V_{22} (F)
		V_1+V_{16} (CB)			$V_{10}+V_{22}$ (CB)
614(0)	619(2)	V_{15} (F)	611(0)	657(81)	V_{18} (F)
		V_6+V_{15} (CB)			V_2+V_{18} (CB)
		$V_{14}+V_{15}$ (CB)			---
613(5)	607(3)	V_{14} (F)	609(0)	646(26)	V_{17} (F)
		V_1+V_{14} (CB)			V_1+V_{17} (CB)
		---			V_2+V_{17} (CB)
612(0)	591(2)	V_{12} (F)	591(6)	584(152)	V_8 (F)
		V_1+V_{12} (CB)			V_1+V_8 (CB)
		---			V_2+V_8 (CB)
592(1.6)	582(7)	V_{13} (F)	588(7)	573(41)	$V_{10}+V_{22}$ (CB)
		V_2+V_{13} (CB)			V_1+V_{10} (CB)

	588(1.7)	571(0.08)	V_{10} (F)	583(13)	559(102)	V_9 (F)
			$V_{10}+V_{22}$ (CB)			V_1+V_9 (CB)
Ring distort	555(0.03)	569(5)	V_{11} (F)	552(0.3)	552(14)	V_6 (F)
			V_1+V_{11} (CB)			V_1+V_6 (CB)
	553(0.02)	568(0.5)	V_9 (F)	551(0.1)	532(0)	V_7 (F)
			V_9+V_{22} (CB)			V_7+V_{27} (CB)
	528(0.02)	557(0.1)	V_8 (F)	526(0.08)	531(24)	V_{16} (F)
			V_1+V_8 (CB)			V_2+V_{16} (CB)
			---			$V_{15}+V_{16}$ (CB)
	510(0)	543(8)	V_6 (F)	508(0)	499(20)	V_{14} (F)
			V_6+V_{17} (CB)			$V_{14}+V_{17}$ (CB)
	505(18)	523(0)	V_4 (F)	497(58)	482(551)	V_{11} (F)
			V_4+V_{11} (CB)			V_1+V_{11} (CB)
			---			$V_{11}+V_{21}$ (CB)
	464(0)	505(0)	V_7 (F)	463(0)	464(31)	V_{12} (F)
			V_7+V_{13} (CB)			V_1+V_{12} (CB)
			---			$V_{12}+V_{21}$ (CB)
	461(0)	481(0)	V_5 (F)	460(0)	461(0)	V_5 (F)
			V_5+V_{21} (CB)			---
	342(0)	337(0)	V_3 (F)	318(0)	380(0)	V_4 (F)
			V_3+V_{44} (CB)			V_4+V_{19} (CB)
	118(0)	84(0)	V_2 (F)	77(0)	168(0)	V_3 (F)
			V_2+V_{49} (CB)			V_3+V_{44} (CB)
	85(0)	57(0)	V_1 (F)	25(0)	82.5(0)	V_2 (F)
			V_1+V_{44} (CB)			V_2+V_{42} (CB)
			V_1+V_{49} (CB)			V_2+V_{49} (CB)

Table S4. The harmonic and anharmonic infrared vibrational frequencies (in cm^{-1}) with relative intensity I (in KM mol^{-1}), including fundamental and combination modes for C_{20} fullerene in the cationic and anionic forms in the gas phase.

C_{20}	Cation in the gas phase			Anion in the gas phase		
Vibrational Modes	Harmonic Freq (Int)	Anharmonic Freq (Int)	Mode	Harmonic Freq (Int)	Anharmonic Freq (Int)	Mode
C-C stretch	1369(0)	1396(0)	V_{54} (F)	1369(0)	1361(0)	V_{54} (F)
			$V_{19}+V_{54}$ (CB)			$V_{17}+V_{54}$ (CB)
	1365(0)	1395(0)	V_{53} (F)	1303(0)	1250(0)	V_{53} (F)

		$V_{42}+V_{53}$ (CB)			$V_{11}+V_{53}$ (CB)
1364(0)	1342(0)	V_{52} (F)	1295(102)	1299(47)	V_{52} (F)
		$V_{48}+V_{52}$ (CB)			$V_{1}+V_{52}$ (CB)
1229(0.9)	1307(0)	V_{51} (F)	1262(0)	1301(0)	V_{51} (F)
		$V_{20}+V_{51}$ (CB)			$V_{33}+V_{51}$ (CB)
1217(1.2)	1301(0)	V_{50} (F)	1246(92)	1254(30)	V_{50} (F)
		$V_{19}+V_{50}$ (CB)			$V_{2}+V_{50}$ (CB)
1216.9(0)	1294(0.08)	V_{49} (F)	1244(10)	1243(262)	V_{49} (F)
		$V_{1}+V_{49}$ (CB)			$V_{1}+V_{49}$ (CB)
1216.8(1.2)	1266(0.6)	V_{48} (F)	1207(0)	1232(0)	V_{48} (F)
		$V_{2}+V_{48}$ (CB)			$V_{39}+V_{48}$ (CB)
1216.5(0)	1265(0.5)	V_{47} (F)	1199(0)	1218(0.09)	V_{47} (F)
		$V_{1}+V_{47}$ (CB)			$V_{1}+V_{47}$ (CB)
1214(0)	1254(0)	V_{46} (F)	1195(10)	1191(62)	V_{46} (F)
		$V_{20}+V_{46}$ (CB)			$V_{2}+V_{46}$ (CB)
1208(0)	1250(0)	V_{45} (F)	1193(0)	1190(0.8)	V_{44} (F)
		$V_{10}+V_{45}$ (CB)			$V_{1}+V_{44}$ (CB)
1197.2(0)	1220(0.4)	V_{44} (F)	1173(19)	1185(0)	V_{45} (F)
		$V_{22}+V_{44}$ (CB)			$V_{11}+V_{45}$ (CB)
1197(0)	1205(0)	V_{43} (F)	1172(0)	1178(8)	V_{40} (F)
		$V_{19}+V_{43}$ (CB)			$V_{40}+V_{44}$ (CB)
		$V_{20}+V_{43}$ (CB)			---
1183(6.4)	1203(9)	V_{42} (F)	1169(0)	1164.5(0)	V_{42} (F)
		$V_{42}+V_{43}$ (CB)			$V_{1}+V_{42}$ (CB)
1182(6.3)	1198(10)	V_{41} (F)	1167(89)	1164.5(0.3)	V_{39} (F)
		$V_{41}+V_{43}$ (CB)			$V_{39}+V_{48}$ (CB)
1174(0.01)	1182(0)	V_{40} (F)	1152(0)	1159(25)	V_{41} (F)
		$V_{18}+V_{40}$ (CB)			$V_{1}+V_{41}$ (CB)
1165(6.4)	1159(0.6)	V_{38} (F)	1144(8)	1145(0)	V_{43} (F)
		$V_{22}+V_{38}$ (CB)			$V_{36}+V_{43}$ (CB)
1164(6.5)	1156(0.6)	V_{39} (F)	1119(33)	1129(14)	V_{38} (F)
		$V_{22}+V_{39}$ (CB)			$V_{2}+V_{38}$ (CB)
1134(5)	1136(0)	V_{36} (F)	1112(0)	1117(1)	V_{35} (F)
		$V_{17}+V_{36}$ (CB)			$V_{5}+V_{35}$ (CB)
		$V_{34}+V_{36}$ (CB)			---

	1121(0)	1127(0)	V_{37} (F)	1109(0.4)	1114(0)	V_{37} (F)
			V_7+V_{37} (CB)			$V_{17}+V_{37}$ (CB)
	1093.6(4)	1107(0.07)	V_{32} (F)	---	---	---
			V_1+V_{32} (CB)	---	---	---
CCC bend	1093.5(0)	1106(0)	V_{34} (F)	1087(1.5)	1109(0.05)	V_{36} (F)
			V_8+V_{34} (CB)			V_1+V_{36} (CB)
						V_3+V_{36} (CB)
	1093(0)	1105(0.2)	V_{33} (F)	1083(0)	1086(0)	V_{34} (F)
			V_2+V_{33} (CB)			$V_{21}+V_{34}$ (CB)
	1092(4)	1104(0)	V_{35} (F)	1022(3)	1045(0.8)	V_{33} (F)
			V_8+V_{35} (CB)			V_2+V_{33} (CB)
	1053(0)	1037(1.4)	V_{31} (F)	1001(0)	983(0)	V_{32} (F)
			$V_{31}+V_{50}$ (CB)			$V_{32}+V_{41}$ (CB)
	1019(0)	981(0)	V_{30} (F)	936(0)	944(0)	V_{29} (F)
			$V_{20}+V_{30}$ (CB)			$V_{20}+V_{29}$ (CB)
	1018(0)	977(0)	V_{29} (F)	932(0)	936(0)	V_{31} (F)
			$V_{19}+V_{29}$ (CB)			V_8+V_{31} (CB)
			---			$V_{31}+V_{33}$ (CB)
	896(3)	895(4)	V_{28} (F)	885(0)	909(0)	V_{30} (F)
			$V_{28}+V_{52}$ (CB)			$V_{20}+V_{30}$ (CB)
	895(2)	894(3)	V_{27} (F)	873(29)	893(33)	V_{28} (F)
			V_4+V_{27} (CB)			V_3+V_{28} (CB)
	880(5)	866(8)	V_{26} (F)	854(31)	877(27)	V_{27} (F)
			V_1+V_{26} (CB)			V_4+V_{27} (CB)
	808(0)	808(0)	V_{25} (F)	848(39)	866(43)	V_{26} (F)
			$V_{25}+V_{48}$ (CB)			V_5+V_{26} (CB)
	750.7(0)	770(0)	V_{24} (F)	787(0)	807(0)	V_{25} (F)
			V_8+V_{24} (CB)			$V_{11}+V_{25}$ (CB)
	750(0)	769(0)	V_{23} (F)	751(0)	768(0)	V_{24} (F)
			$V_{23}+V_{38}$ (CB)			$V_{12}+V_{24}$ (CB)
	738(0)	758(0)	V_{22} (F)	746(0)	761(0)	V_{23} (F)
			$V_{22}+V_{39}$ (CB)			$V_{11}+V_{23}$ (CB)
	737(0)	746(0)	V_{21} (F)	706(0)	722(0)	V_{22} (F)
			$V_{14}+V_{21}$ (CB)			V_9+V_{22} (CB)
	712(0)	679(0.2)	V_{19} (F)	705(92)	699(58)	V_{21} (F)

			$V_{17}+V_{19}$ (CB)			$V_{10}+V_{21}$ (CB)
	711(0)	677(0.2)	V_{20} (F)	679(4)	685(0.5)	V_{20} (F)
			$V_{20}+V_{46}$ (CB)			$V_{1}+V_{20}$ (CB)
	682(2)	667(0.8)	V_{18} (F)	664(0)	625(0)	V_{19} (F)
			$V_{18}+V_{40}$ (CB)			$V_{12}+V_{19}$ (CB)
			---			$V_{19}+V_{39}$ (CB)
			---			$V_{17}+V_{37}$ (CB)
	646(0.8)	656(0)	V_{17} (F)	639(0)	623(0.05)	V_{16} (F)
			$V_{9}+V_{17}$ (CB)			$V_{10}+V_{16}$ (CB)
			$V_{17}+V_{19}$ (CB)			---
	645(0.8)	645(0)	V_{16} (F)	635(16)	621(3)	V_{15} (F)
			$V_{8}+V_{16}$ (CB)			$V_{10}+V_{15}$ (CB)
	620(0.06)	611(0.7)	V_{15} (F)	619(0.6)	599(12)	V_{14} (F)
			$V_{15}+V_{43}$ (CB)			$V_{3}+V_{14}$ (CB)
	619(0.08)	610(1)	V_{14} (F)	616(5)	572.5(0.4)	$V_{11}+V_{53}$ (CB)
			$V_{14}+V_{52}$ (CB)			$V_{11}+V_{19}$ (CB)
			$V_{14}+V_{45}$ (CB)			---
	590(4)	608(0.1)	V_{13} (F)	583(2)	572(0.3)	V_{11} (F)
			$V_{3}+V_{13}$ (CB)			$V_{2}+V_{11}$ (CB)
			$V_{13}+V_{46}$ (CB)			$V_{11}+V_{19}$ (CB)
	589(1.6)	586(0.2)	V_{12} (F)	582(9)	571(12)	V_{13} (F)
			$V_{2}+V_{12}$ (CB)			$V_{13}+V_{19}$ (CB)
			$V_{11}+V_{43}$ (CB)			---
	554(0)	585(0.2)	V_{11} (F)	558(4)	562(0)	V_{18} (F)
			$V_{2}+V_{11}$ (CB)			$V_{9}+V_{18}$ (CB)
			$V_{11}+V_{43}$ (CB)			$V_{15}+V_{18}$ (CB)
	542(0.3)	568(0.002)	V_{10} (F)	555.5(4.5)	557(18)	V_{12} (F)
			$V_{10}+V_{45}$ (CB)			$V_{1}+V_{12}$ (CB)
Ring distort	541(0.3)	567(0.01)	V_{9} (F)	555(0)	542(6)	V_{9} (F)
			$V_{9}+V_{17}$ (CB)			$V_{9}+V_{18}$ (CB)
	527(0)	564(0.002)	V_{8} (F)	533(0.7)	527(4)	V_{8} (F)
			$V_{8}+V_{16}$ (CB)			$V_{1}+V_{8}$ (CB)
			$V_{8}+V_{35}$ (CB)			$V_{8}+V_{18}$ (CB)
	523(2)	549(3)	V_{7} (F)	509(9)	510(9)	V_{7} (F)
			$V_{2}+V_{7}$ (CB)			$V_{1}+V_{2}$ (CB)

522(0)	533(0)	V_6 (F)	483(17)	479(0)	V_{10} (F)
		V_6+V_{49} (CB)			$V_{10}+V_{15}$ (CB)
		---			$V_{10}+V_{16}$ (CB)
521(0)	495(0)	V_5 (F)	475.8(0)	463(0)	V_6 (F)
		V_5+V_{20} (CB)			V_6+V_{20} (CB)
469(0)	492(0)	V_4 (F)	395(0)	299(0)	V_5 (F)
		V_4+V_{19} (CB)			V_5+V_{39} (CB)
		---			V_5+V_{49} (CB)
411(0)	453(0)	V_3 (F)	389(0)	266(0)	V_4 (F)
		V_3+V_{49} (CB)			V_4+V_{27} (CB)
308(0)	379(0)	V_2 (F)	262(0)	252(0)	V_3 (F)
		V_2+V_7 (CB)			V_3+V_{49} (CB)
306(0)	377(0)	V_1 (F)	196(0)	138(0)	V_2 (F)
		V_1+V_{26} (CB)			V_2+V_{33} (CB)
---	---	---	169(0)	140(0)	V_1 (F)
		---			V_1+V_{49} (CB)

Table S5. The harmonic and anharmonic infrared vibrational frequencies (in cm^{-1}) with relative intensity I (in KM mol^{-1}), including fundamental and combination modes for neutral $N_{10}C_{10}$ heterofullerene in the gas and water solvent states.

$N_{10}C_{10}$	Neutral in the gas phase			Neutral in water solvent		
Vibrational Modes	Harmonic Freq (Int)	Anharmonic Freq (Int)	Mode	Harmonic Freq (Int)	Anharmonic Freq (Int)	Mode
C-C stretch	1499.7(0)	1517(0.002)	V_{54} (F)	1506(0.002)	1525(0)	V_{54} (F)
			V_1+V_{54} (CB)	1506(0.002)		V_1+V_{54} (CB)
	1499(0)	1516(0.001)	V_{53} (F)	1480(0.001)	1524(0)	V_{53} (F)
			V_1+V_{53} (CB)	1480(0.001)		V_1+V_{53} (CB)
	1475.6(0)	1493(0)	V_{51} (F)	---	1497(0.3)	V_{52} (F)
			$V_{20}+V_{51}$ (CB)			$V_{21}+V_{52}$ (CB)
	1475(0)	1492(0)	V_{52} (F)	---	1496(0.4)	V_{51} (F)
			$V_{20}+V_{52}$ (CB)			$V_{21}+V_{51}$ (CB)
	1403(0)	1405(0)	V_{50} (F)	1403(0)	1411(0)	V_{50} (F)
			V_1+V_{50} (CB)			V_1+V_{50} (CB)
	1369(0)	1382(0.001)	V_{48} (F)	---	1391(0)	V_{49} (F)
			$V_{11}+V_{48}$ (CB)			$V_{14}+V_{49}$ (CB)
			---		1391(0)	V_{48} (F)

			---			$V_{13}+V_{48}$ (CB)
	1368(0)	1381(0)	V_{49} (F)	1373(0)	1347(4)	V_{47} (F)
			$V_{12}+V_{49}$ (CB)			$V_{22}+V_{47}$ (CB)
			---	1373(0)	1347(4)	V_{46} (F)
			---			$V_{21}+V_{46}$ (CB)
	1325(6.5)	1346(2)	V_{47} (F)	1330(17)	1257(0.009)	V_{45} (F)
	1325(6.5)		$V_{38}+V_{47}$ (CB)	1330(17)		$V_{21}+V_{45}$ (CB)
	1201(0)	1345(3)	V_{46} (F)	1210(0.03)	1002(0)	V_{44} (F)
			$V_{20}+V_{46}$ (CB)			$V_{3}+V_{44}$ (CB)
			---		1002(0)	V_{43} (F)
			---			$V_{43}+V_{52}$ (CB)
C-N stretch	988(0)	1251(0)	V_{45} (F)	992(0)	936(0.003)	V_{42} (F)
			$V_{20}+V_{45}$ (CB)	992(0)		$V_{5}+V_{42}$ (CB)
	987(0)	985(0)	V_{43} (F)	927(0.003)	935(0.002)	V_{41} (F)
			$V_{2}+V_{43}$ (CB)	927(0.003)		$V_{4}+V_{41}$ (CB)
	919.4(0)	984(0)	V_{44} (F)	916(0.6)	929.6(1.1)	V_{40} (F)
			$V_{2}+V_{44}$ (CB)	916(0.6)		$V_{21}+V_{40}$ (CB)
	919(0)	926.8(0)	V_{42} (F)	888(0)	929(1)	V_{39} (F)
		926.8(0)	$V_{5}+V_{42}$ (CB)	888(0)		$V_{22}+V_{39}$ (CB)
			V_{41} (F)			---
			$V_{4}+V_{41}$ (CB)		---	
	912(0.2)	925.8(0.1)	V_{40} (F)	875(0.4)	898(0)	V_{37} (F)
	912(0.2)		$V_{20}+V_{40}$ (CB)			$V_{37}+V_{45}$ (CB)
	879(0.001)	925(0.07)	V_{39} (F)	811(0)	895(2)	V_{38} (F)
			$V_{21}+V_{39}$ (CB)	811(0)	895(2)	$V_{8}+V_{38}$ (CB)
			---			V_{36} (F)
			---			$V_{36}+V_{54}$ (CB)
	878(0)	891(0.08)	V_{38} (F)	---	800(0)	V_{31}
			$V_{38+}V_{45}$ (CB)			$V_{31}+V_{35}$ (CB)
	863(2)	886(0.06)	V_{37} (F)	---	836(0.006)	V_{35} (F)
			$V_{37}+V_{45}$ (CB)			$V_{11}+V_{35}$ (CB)
CNN/CCN bend	807(0)	859(5)	V_{36} (F)	790(0)	842(0.004)	V_{34} (F)
			$V_{15}+V_{36}$ (CB)	790(0)		$V_{11}+V_{34}$ (CB)

	806.7(0)	837(0)	V_{35} (F)	780(0)	799(0)	V_{30} (F)
			V_{14}+V_{35} (CB)	780(0)		V_{30}+V_{34} (CB)
	789(0)	836(0)	V_{34} (F)	749(0)	793(0.003)	V_{33} (F)
			V_{14}+V_{34} (CB)	749(0)		V_{1}+V_{33} (CB)
			---		793(0.003)	V_{32} (F)
			---			V_{1}+V_{32} (CB)
	788(0)	797(0.001)	V_{31} (F)	746(0)	751(0.001)	V_{27} (F)
		797(0.001)	V_{3}+V_{31} (CB)			V_{1}+V_{27} (CB)
			V_{30} (F)			---
			V_{11}+V_{30} (CB)			---
	773(0)	793(0.008)	V_{33} (F)	720(101)	726(71)	V_{26} (F)
		793(0.008)	V_{1}+V_{33} (CB)			V_{17}+V_{26} (CB)
			V_{32} (F)	720(101)	726(71)	V_{25} (F)
			V_{1}+V_{32} (CB)			V_{17}+V_{25} (CB)
	772(0)	745(0)	V_{27} (F)	---	722(0.002)	V_{29} (F)
			V_{1}+V_{27} (CB)			V_{1}+V_{29} (CB)
	748.4(0)	734(25)	V_{25} (F)	---	720.5(0.009)	V_{28} (F)
			V_{25}+V_{50} (CB)			V_{11}+V_{28} (CB)
	748(0)	733(26)	V_{26} (F)	---	701(0.003)	V_{24} (F)
			V_{26}+V_{50} (CB)			V_{14}+V_{24} (CB)
			---		701(0.003)	V_{23} (F)
			---			V_{13}+V_{23} (CB)
	742(0)	722(0.002)	V_{28} (F)	---	673(0.002)	V_{22} (F)
			V_{13}+V_{28} (CB)			V_{22}+V_{45} (CB)
		722(0.002)	V_{29} (F)			
			V_{14}+V_{29} (CB)			
CCC bend	723(41)	701(0.01)	V_{23} (F)	695(0)	668(0.001)	V_{21} (F)
	723(41)		V_{11}+V_{23} (CB)	695(0)		V_{21}+V_{45} (CB)
	693(0.01)	700(0.01)	V_{24} (F)			
	693(0.01)	700(0.01)	V_{12}+V_{24} (CB)			
			V_{23} (F)			---
			V_{11}+V_{23} (CB)			
	657(0)	671(0)	V_{22} (F)			---

			$V_{22}+V_{40}$ (CB)			
	652(0.08)	656(0.02)	V_{21} (F)	666(0)	676(0)	V_{20} (F)
			$V_{21}+V_{45}$ (CB)			$V_{20}+V_{40}$ (CB)
	652(0.08)	657(0.02)	V_{20} (F)	665(0)	633(0.05)	V_{19} (F)
			$V_{20}+V_{45}$ (CB)			$V_{19}+V_{37}$ (CB)
Ring distort	621(0.5)	625(0.01)	V_{17} (F)	661(0)	632(0.04)	V_{18} (F)
			$V_{1}+V_{17}$ (CB)			$V_{18}+V_{38}$ (CB)
	620(0.5)	624(0.006)	V_{19} (F)	628(2)	621(0)	V_{17} (F)
	620(0.5)		$V_{19}+V_{38}$ (CB)	628(2)		$V_{1}+V_{17}$ (CB)
	603(14)	623(0.004)	V_{18} (F)	618(0)	595(23)	V_{16} (F)
			$V_{18}+V_{20}$ (CB)			$V_{16}+V_{53}$ (CB)
	563(0)	592(9)	V_{16} (F)	605(37)	532(0.006)	V_{13} (F)
			$V_{16}+V_{53}$ (CB)			$V_{13}+V_{48}$ (CB)
	515(0.05)	590(0.006)	V_{15} (F)	564(0)	530(0.08)	V_{14}
	515(0.05)		$V_{9}+V_{15}$ (CB)			$V_{5}+V_{14}$ (CB)
			$V_{10}+V_{15}$ (CB)			---
	512(0)	525(0.007)	V_{11} (F)	523(0.005)	507(0)	V_{15} (F)
			$V_{11}+V_{48}$ (CB)	523(0.005)		$V_{15}+V_{36}$ (CB)
	511(0)	524(0.004)	V_{12} (F)	522(0.004)	487(0.004)	V_{12} (F)
			$V_{12}+V_{14}$ (CB)			$V_{12}+V_{34}$ (CB)
			---	522(0.004)	487(0.004)	V_{11} (F)
			---			$V_{11}+V_{34}$ (CB)
	493(0)	495(0.05)	V_{10} (F)	502(0.03)	504(0.2)	V_{10} (F)
			$V_{10}+V_{21}$ (CB)			$V_{10}+V_{38}$ (CB)
	492(0)	496(0.06)	V_{9} (F)	502(0.03)	504(0.2)	V_{9} (F)
			$V_{9}+V_{20}$ (CB)			$V_{9}+V_{21}$ (CB)
	463(0)	486(0.3)	V_{14} (F)	467(0.003)	483(0.002)	V_{8} (F)
			$V_{14}+V_{3}$ (CB)			$V_{8}+V_{21}$ (CB)
	447(0)	484(2)	V_{13} (F)	450(0)	454(0)	V_{7} (F)
			$V_{13}+V_{34}$ (CB)			$V_{7}+V_{45}$ (CB)
	446(0)	479(0)	V_{8} (F)	450(0)	453(0)	V_{6} (F)
			$V_{8}+V_{20}$ (CB)			$V_{6}+V_{45}$ (CB)
	344(0.02)	452(0.001)	V_{6} (F)	351(0.004)	353.4(0.003)	V_{4} (F)

			V_1+V_6 (CB)			V_4+V_{41} (CB)
		452(0.001)	V_7 (F)			---
			V_1+V_7 (CB)			---
	342(0)	349(0.004)	V_4 (F)	351(0.004)	353(0.03)	V_5 (F)
			V_4+V_{11} (CB)			V_4+V_{41} (CB)
	336(0)	347(0.1)	V_5 (F)	339(0.2)	327(301)	V_1 (F)
			V_5+V_{52} (CB)			V_1+V_{54} (CB)
	335(0)	329(161)	V_1 (F)	339(0.2)	309(0.6)	V_3 (F)
			V_1+V_{54} (CB)			V_3+V_{44} (CB)
	264(211)	306(0.002)	V_2 (F)	289(468)	307(0.02)	V_2 (F)
			V_2+V_{43} (CB)			V_2+V_{44} (CB)

Table S6. The harmonic and anharmonic infrared vibrational frequencies (in cm^{-1}) with relative intensity I (in KM mol^{-1}), including fundamental and combination modes for $N_{10}C_{10}$ heterofullerene in the cationic and anionic forms in the gas phase.

$N_{10}C_{10}$	Cation in the gas phase			Anion in the gas phase		
Vibrational Modes	Harmonic Freq (Int)	Anharmonic Freq (Int)	Mode	Harmonic Freq (Int)	Anharmonic Freq (Int)	Mode
C-C stretch	1464(32)	1775(14)	V_{54} (F)	1494(0)	1533(50)	V_{52} (F)
			V_5+V_{54} (CB)			V_5+V_{52} (CB)
			---			$V_{48}+V_{52}$ (CB)
	1454(20)	1774(2)	V_{53} (F)	1470(2)	1474(199)	V_{51} (F)
			V_5+V_{53} (CB)			V_1+V_{51} (CB)
	1432(1)	1757(15)	V_{52} (F)	1377(0.2)	1357(50)	V_{47} (F)
			V_5+V_{52} (CB)			V_1+V_{47} (CB)
	1411(12)	1737(47)	V_{51} (F)	1376(5)	1371(0.006)	V_{45} (F)
			$V_{33}+V_{51}$ (CB)			V_1+V_{45} (CB)
			$V_{41}+V_{51}$ (CB)			$V_{46}+V_{52}$ (CB)
	1347(1.5)	1684(4)	V_{50} (F)	1359(0)	1294(134)	V_{46} (F)
			V_5+V_{50} (CB)			V_1+V_{46} (CB)
	1334(17)	1656(4)	V_{49} (F)	1328(0)	1270(322)	V_{53} (F)
			V_6+V_{49} (CB)			V_1+V_{53} (CB)
	1313(0.02)	1642(165)	V_{48} (F)	1325(73)	1261(128)	V_{50} (F)
			V_1+V_{48} (CB)			V_5+V_{50} (CB)
	1290(0.4)	1573(0.2)	V_{47} (F)	1275(17)	1153(0.4)	V_{49} (F)

			V_2+V_{47} (CB)			V_5+V_{49} (CB)
		1573(0.2)	V_{46} (F)			---
			V_2+V_{46} (CB)			---
		1573(0.2)	V_{45} (F)			---
			V_2+V_{45} (CB)			---
	1289(1.3)	1486(62)	V_{44} (F)	1254(0.07)	1150(127)	V_{48} (F)
			V_1+V_{44} (CB)			V_1+V_{48} (CB)
			$V_{33}+V_{44}$ (CB)			$V_{48}+V_{52}$ (CB)
	1264(16)	1406(5)	V_{43} (F)	1232(0)	1087(2)	V_{54} (F)
			$V_{30}+V_{43}$ (CB)			V_5+V_{54} (CB)
	1045(0.02)	1368(14)	V_{42} (F)	---	1054(0.4)	V_{49} (F)
			V_3+V_{42} (CB)			V_5+V_{49} (CB)
	1028(0.6)	1346(30)	V_{41} (F)	---	1002(0.01)	V_{44} (F)
			$V_{30}+V_{41}$ (CB)			V_5+V_{44} (CB)
			$V_{41}+V_{44}$ (CB)			---
			$V_{41}+V_{48}$ (CB)			---
C-N stretch	985(0.5)	1300(3)	V_{39} (F)	991(10)	999(0.001)	V_{43} (F)
			V_1+V_{39} (CB)	991(10)		V_4+V_{43} (CB)
	971(5)	1297(14)	V_{40} (F)	944(0.4)	963(0.3)	V_{36} (F)
			$V_{30}+V_{40}$ (CB)			V_7+V_{36} (CB)
	953(2)	1288(5)	V_{38} (F)	931(0.1)	948(8)	V_{42} (F)
			$V_{30}+V_{38}$ (CB)			V_6+V_{46} (CB)
	926(3)	1250(5)	V_{37} (F)	909(0.004)	943(2)	V_{41} (F)
			V_1+V_{37} (CB)			V_3+V_{41} (CB)
	903(3)	1247(21)	V_{36} (F)	907(3.5)	914(9)	V_{37} (F)
			$V_{36}+V_{44}$ (CB)			$V_{10}+V_{37}$ (CB)
	888(6)	1231(138)	V_{35} (F)	902(3)	911(19)	V_{38} (F)
			V_2+V_{35} (CB)			V_1+V_{38} (CB)
			---			V_8+V_{38} (CB)
	887(0.3)	1205(331)	V_{33} (F)	896(0.3)	900(19)	V_{39} (F)
			$V_{33}+V_{48}$ (CB)			$V_{10}+V_{39}$ (CB)
			$V_{33}+V_{44}$ (CB)			---
	844(2)	1199(10)	V_{34} (F)	856(0)	832(16)	V_{34} (F)

			$V_{1}+V_{34}$ (CB)			$V_{1}+V_{34}$ (CB)
			$V_{2}+V_{34}$ (CB)			$V_{10}+V_{34}$ (CB)
	824(3)	1139(1)	V_{32} (F)	---	---	---
			$V_{30}+V_{41}$ (CB)			---
			$V_{30}+V_{33}$ (CB)			---
			$V_{30}+V_{12}$ (CB)			
	823(1)	1097(17)	V_{31} (F)	---	---	---
			$V_{6}+V_{31}$ (CB)			---
			$V_{11}+V_{31}$ (CB)			
	781(0.1)	1067(10)	V_{30} (F)	---	---	---
			$V_{30}+V_{41}$ (CB)		---	---
			$V_{30}+V_{33}$ (CB)			---
			$V_{12}+V_{30}$ (CB)			---
CNN/CCN bend	769(15)	1057(3)	V_{29} (F)	800(2)	823(71)	V_{35} (F)
			$V_{29}+V_{44}$ (CB)			$V_{1}+V_{35}$ (CB)
	762(3)	1045(1)	V_{28} (F)	799(3)	805(0.04)	V_{33} (F)
			$V_{28}+V_{41}$ (CB)			$V_{12}+V_{33}$ (CB)
	759(16)	949(19)	V_{27} (F)	786(0)	790(0.08)	V_{32} (F)
			$V_{27}+V_{44}$ (CB)			$V_{9}+V_{32}$ (CB)
			$V_{19}+V_{27}$ (CB)			---
	752(4)	931(8)	V_{26} (F)	780(0)	776(0.09)	V_{31} (F)
			$V_{26}+V_{4}$ (CB)			---
			$V_{17}+V_{26}$ (CB)			$V_{13}+V_{31}$ (CB)
	742(20)	892(7)	V_{25} (F)	774(0)	769(0.04)	V_{22} (F)
			$V_{1}+V_{25}$(CB)			$V_{22}+V_{27}$ (CB)
CCC bend	738(13)	865(10)	V_{24} (F)	764(0.009)	766(0.06)	V_{30} (F)
			$V_{24}+V_{48}$ (CB)			$V_{16}+V_{30}$ (CB)
	729(6)	860(3)	V_{23} (F)	737(0.005)	750(0.016)	V_{29} (F)
			$V_{7}+V_{23}$ (CB)			$V_{5}+V_{29}$ (CB)
	728(0.2)	847(2)	V_{22} (F)	720(16)	733(0.02)	V_{21} (F)
			$V_{9}+V_{22}$ (CB)			$V_{21}+V_{27}$ (CB)
	719(0.2)	838(1.2)	V_{21} (F)	712(62)	721(0.06)	V_{40} (F)
			$V_{10}+V_{21}$ (CB)			$V_{7}+V_{40}$ (CB)

			V_9+V_{21} (CB)			---
			$V_{17}+V_{26}$ (CB)			---
	675(2.4)	822(0.1)	V_{19} (F)	708(46)	720(768)	V_{26} (F)
			$V_{19}+V_{27}$ (CB)			V_1+V_{26} (CB)
			---			$V_{18}+V_{26}$ (CB)
	669(3)	816(0.2)	V_{20} (F)	706(19)	702(216)	V_{27} (F)
			$V_{17}+V_{20}$ (CB)			V_1+V_{27} (CB)
			---			$V_{17}+V_{27}$ (CB)
	658(2.3)	802(0.2)	V_{18} (F)	681(0.004)	695(99)	V_{25} (F)
			V_6+V_{18} (CB)			$V_{30}+V_{25}$ (CB)
			---			V_1+V_{25} (CB)
	656(0.4)	794(3)	V_{17} (F)	663(0.003)	688(245)	V_{28} (F)
			$V_{17}+V_{20}$ (CB)			V_1+V_{28} (CB)
			$V_{17}+V_{26}$ (CB)			---
	---	---	---	660(0.004)	677(0.02)	V_{24} (F)
			---	660(0.004)		V_9+V_{24} (CB)
	---	---	---	---	654(0.4)	V_8 (F)
			---			V_4+V_8 (CB)
			---			V_2+V_8 (CB)
Ring distort	636(0.2)	784(0.9)	V_{16} (F)	622(6)	629(27)	V_{19} (F)
			$V_{16}+V_{19}$ (CB)			V_1+V_{19} (CB)

	617(8)	764(0.5)	V_{15} (F)	621(0.8)	614(0.04)	V_{18} (F)
			$V_{13}+V_{15}$ (CB)			$V_{18}+V_{26}$ (CB)
	561(34)	723(6)	V_{14} (F)	608(0)	613(15)	V_{20} (F)
			$V_{17}+V_{14}$ (CB)			V_3+V_{20} (CB)
	560(0.3)	704(2)	V_{13} (F)	560(0.02)	600(0.3)	V_{17} (F)
			$V_{13}+V_{15}$ (CB)			V_5+V_{17} (CB)
	542(0.3)	660(12)	V_{12} (F)	555(15)	561(10)	V_{16} (F)
			$V_{12}+V_{30}$ (CB)			$V_{16}+V_{30}$ (CB)
	529(0.7)	656(1)	V_{11} (F)	513(0)	512(82)	V_{14} (F)
			V_1+V_{11} (CB)			V_1+V_{14} (CB)
			$V_{11}+V_{31}$ (CB)			---

512(12)	627(0.2)	V_{10} (F)	506(1.3)	498(29)	V_{12} (F)
		$V_{10}+V_{21}$ (CB)			V_1+V_{12} (CB)
502(84)	618(0.9)	V_8 (F)	497(16)	479(60)	V_{13} (F)
		V_4+V_8 (CB)			$V_{13}+V_{31}$ (CB)
		V_8+V_{30} (CB)			---
497(10)	613(1)	V_9 (F)	491(2)	438(0.03)	V_{11} (F)
		V_9+V_{21} (CB)			V_2+V_{11} (CB)
		---			V_4+V_{11} (CB)
		---			V_5+V_{11} (CB)
464(5)	606(1.3)	V_7 (F)	472(0.3)	410(0.4)	V_{10} (F)
		V_7+V_{23} (CB)			V_2+V_{10} (CB)
		V_7+V_{30} (CB)			V_4+V_{10} (CB)
459(95)	---	---	461(0.2)		V_1+V_9 (CB)
452(23)	---	---	457(30)	375(133)	V_9 (F)
					V_1+V_9 (CB)
429(4)	564(0.9)	V_6 (F)	443(0.01)	373(6)	V_6 (F)
		V_6+V_{30} (CB)			V_2+V_6 (CB)
		V_6+V_{31} (CB)			V_5+V_6 (CB)
378(2)	537(2)	V_5 (F)	428(22)	361(223)	V_5 (F)
		V_5+V_{33} (CB)			V_5+V_6 (CB)
		---			V_5+V_{52}
363(17)	535(1.2)	V_4 (F)	357(0.6)	253(1)	V_4 (F)
		V_4+V_{26} (CB)			V_4+V_8 (CB)
347(6)	518(5)	V_3 (F)	330(264)	279(1.4)	V_3+V_7 (CB)
		V_3+V_{34} (CB)			V_3+V_7 (CB)
340(85)	412(1.5)	V_2 (F)	291(0.02)	253(1.3)	V_4 (F)
		V_2+V_{35} (CB)			V_4+V_8 (CB)
49(9)	409.4(0.6)	V_1 (F)	281(293)	240(2)	V_{15} (F)
		V_1+V_{11} (CB)			V_4+V_{15} (CB)
---	---	---	254(265)	163(0.8)	V_2 (F)
					V_2+V_6 (CB)
---	---	---	30(31)	79(0.33)	V_1 (F)
					V_1+V_7 (CB)

Table S7. The harmonic and anharmonic infrared vibrational frequencies (in cm^{-1}) with relative intensity I (in KM mol^{-1}), including fundamental and combination modes for neutral $C_{12}N_8$ heterofullerene in the cationic and anionic forms in the gas phase.

$C_{12}N_8$	Neutral in the gas phase			Neutral in water solvent		
Vibrational Modes	Harmonic Freq (Int)	Anharmonic Freq (Int)	Mode	Harmonic Freq (Int)	Anharmonic Freq (Int)	Mode
C-C stretch	1613(0)	1632(0)	V_{54} (F)	1614(0)	1631(0)	V_{54} (F)
			V_6+V_{54} (CB)			V_4+V_{54} (CB)
	1613(0)	1632(0)	V_{53} (F)	1614(0)	1631(0)	V_{53} (F)
			V_4+V_{53} (CB)			V_6+V_{53} (CB)
	1601(31)	1620(14)	V_{52} (F)	1600(61)	1619(23)	V_{52} (F)
			V_2+V_{52} (CB)			V_1+V_{52} (CB)
	1601(31)	1620(14)	V_{51} (F)	1600(61)	1619(23)	V_{51} (F)
			V_1+V_{51} (CB)			V_2+V_{51} (CB)
	1601(31)	1620(14)	V_{50} (F)	1600(61)	1619(23)	V_{50} (F)
			V_3+V_{50} (CB)			V_3+V_{50} (CB)
	1575(0)	1591(0)	V_{49} (F)	1576(0)	1590(0)	V_{49} (F)
			V_6+V_{49} (CB)			V_4+V_{49} (CB)
	1138(0)	1146(0)	V_{48} (F)	1137(0)	1145(0)	V_{48} (F)
			V_5+V_{48} (CB)			V_4+V_{48} (CB)
	1138(0)	1146(0)	V_{47} (F)	1137(0)	1145(0)	V_{47} (F)
			V_4+V_{47} (CB)			V_4+V_{47} (CB)
	1138(0)	1146(0)	V_{46} (F)	1137(0)	1145(0)	V_{46} (F)
			V_6+V_{46} (CB)			V_5+V_{46} (CB)
	1121(0)	1135(0)	V_{45} (F)	1118(0)	1117(0)	V_{45} (F)
			$V_{32}+V_{45}$ (CB)			$V_{31}+V_{45}$ (CB)
	1083(0)	1089(0)	V_{44} (F)	1084(0)	1090(0)	V_{44} (F)
			V_2+V_{44} (CB)			V_1+V_{44} (CB)
	1083(0)	1089(0)	V_{43} (F)	1084(0)	1090(0)	V_{43} (F)
			$V_{39}+V_{43}$ (CB)			V_2+V_{43} (CB)
C-N stretch	1059(92)	1071(35)	V_{42} (F)	1057(191)	1062(56)	V_{42} (F)
			$V_{39}+V_{42}$ (CB)			$V_{38}+V_{42}$ (CB)
	1059(92)		---	1057(191)	1062(56)	V_{41} (F)
			---			$V_{39}+V_{41}$ (CB)

	1059(92)		---	1057(191)	1062(56)	V_{40} (F)
			---			$V_{37}+V_{40}$ (CB)
	1002(0)	1069(42)	V_{41} (F)	1003(0)	1008(1.5)	V_{37} (F)
			$V_{37}+V_{41}$ (CB)			$V_{37}+V_{43}$ (CB)
	1002(0)	1069(42)	V_{40} (F)	1003(0)	1008(1.5)	V_{38} (F)
			$V_{14}+V_{40}$ (CB)			$V_{10}+V_{38}$ (CB)
	1002(0)		---	1003(0)	1008(1.5)	V_{39} (F)
			---			$V_{11}+V_{39}$ (CB)
	926(8)	1004(0)	V_{39} (F)	926(19)	938(5)	V_{36} (F)
			$V_{9}+V_{39}$ (CB)			$V_{18}+V_{36}$ (CB)
	926(8)	1004(0)	V_{38} (F)	926(19)	938(5)	V_{35} (F)
			$V_{11}+V_{38}$ (CB)			$V_{17}+V_{35}$ (CB)
	926(8)	1004(0)	V_{37} (F)	926(19)	938(5)	V_{34} (F)
			$V_{10}+V_{37}$ (CB)			$V_{16}+V_{34}$ (CB)
	885(0)	938(3)	V_{36} (F)	886(0)	899(0.1)	V_{33} (F)
			$V_{1}+V_{36}$ (CB)			$V_{25}+V_{33}$ (CB)
	885(0)	938(3)	V_{35} (F)	886(0)	899(0.1)	V_{32} (F)
			$V_{2}+V_{35}$ (CB)			$V_{23}+V_{32}$ (CB)
	885(0)	938(3)	V_{34} (F)	886(0)	899(0.1)	V_{31} (F)
			$V_{3}+V_{34}$ (CB)			$V_{24}+V_{31}$ (CB)
	827(0)	898(0)	V_{33} (F)	828(0)	837(0.1)	V_{29} (F)
			$V_{25}+V_{33}$ (CB)			$V_{29}+V_{35}$ (CB)
	827(0)	898(0)	V_{32} (F)	828(0)	837(0.1)	V_{30} (F)
			$V_{24}+V_{32}$ (CB)			$V_{30}+V_{36}$ (CB)
	827(0)	898(0)	V_{31} (F)	---		---
			$V_{23}+V_{31}$ (CB)			---
CNN bend	748(8)	835(0)	V_{30} (F)	749(19)	761(11)	V_{28} (F)
			$V_{28}+V_{30}$ (CB)			$V_{30}+V_{36}$ (CB)
	748(8)	835(0)	V_{29} (F)	749(19)	761(11)	V_{27} (F)
			$V_{29}+V_{34}$ (CB)			$V_{27}+V_{31}$ (CB)
			---	749(19)	761(11)	V_{26} (F)
			---			$V_{26}+V_{32}$ (CB)
	726(28)	759(5.6)	V_{28} (F)	723(64)	733(0)	V_{21} (F)

			V_1+V_{28} (CB)			$V_{21}+V_{52}$ (CB)
	726(28)	759(5.6)	V_{27} (F)	723(64)	733(0)	V_{20} (F)
			V_3+V_{27} (CB)			$V_{20}+V_{50}$ (CB)
	726(28)	759(5.6)	V_{26} (F)	723(64)	733(0)	V_{19} (F)
			V_2+V_{26} (CB)			$V_{19}+V_{51}$ (CB)
	723(0)	731(0)	V_{21} (F)	722(0)	728(52)	V_{25} (F)
			$V_{21}+V_{52}$ (CB)			$V_{25}+V_{33}$ (CB)
	723(0)	731(0)	V_{20} (F)		728(52)	V_{24} (F)
			$V_{20}+V_{51}$ (CB)			$V_{24}+V_{31}$ (CB)
	723(0)	731(0)	V_{19} (F)		728(52)	V_{23} (F)
			$V_{19}+V_{50}$ (CB)			$V_{23}+V_{32}$ (CB)
	716(0)	730(29)	V_{25} (F)	716.4(0)	718(0)	V_{18} (F)
			V_1+V_{25} (CB)			$V_{18}+V_{52}$ (CB)
	716(0)	730(29)	V_{24} (F)	716.4(0)	718(0)	V_{17} (F)
			V_3+V_{24} (CB)			$V_{17}+V_{51}$ (CB)
	716(0)	730(29)	V_{23} (F)	716.4(0)	718(0)	V_{16} (F)
			$V_{23}+V_{31}$ (CB)			$V_{16}+V_{50}$ (CB)
	708(0)	720(0)	V_{18} (F)	707(0)	707(25)	V_{15} (F)
			$V_{18}+V_{52}$ (CB)			V_2+V_{15} (CB)
	708(0)	720(0)	V_{17} (F)	707(0)	---	V_3+V_{15} (CB)
			$V_{17}+V_{51}$ (CB)			$V_{37}+V_{15}$ (CB)
	708(0)	720(0)	V_{16} (F)	707(0)	---	$V_{38}+V_{15}$ (CB)
			$V_{16}+V_{15}$ (CB)			$V_{39}+V_{15}$ (CB)
	692(0)	703(0)	V_{15} (F)	695(0)	699(0)	V_{14} (F)
			V_3+V_{15} (CB)			V_4+V_{14} (CB)
	676(0)	686(0)	V_{14} (F)	680(0)	644(0)	V_{22} (F)
			V_6+V_{14} (CB)			V_6+V_{22} (CB)
	591(0)	603(0)	V_{22} (F)	592(0)	---	---
			V_6+V_{22} (CB)	592(0)		
Ring distort	574(21)	602(0)	V_{13} (F)	573(46)	603(16)	V_{13} (F)
			$V_{13}+V_{39}$ (CB)	573(46)		$V_{13}+V_{37}$ (CB)
	574(21)	602(0)	V_{12} (F)	573(46)	603(16)	V_{12} (F)
			$V_{12}+V_{37}$ (CB)			$V_{12}+V_{38}$ (CB)

	478(0)	583(22)	V_{11} (F)	478.5(0)	582.8(36)	V_{11} (F)
			V_7+V_{11} (CB)			V_7+V_{11} (CB)
	478(0)	583(22)	V_{10} (F)	478.5(0)	582.8(36)	V_{10} (F)
			V_8+V_{10} (CB)			V_7+V_{10} (CB)
	478(0)	583(22)	V_9 (F)	---	582.8(36)	V_9 (F)
			V_7+V_9 (CB)			V_8+V_9 (CB)
	432(101)	483(0)	V_8 (F)	426(230)	484(0)	V_8 (F)
			V_8+V_{10} (CB)			V_8+V_{10} (CB)
	432(101)	483(0)	V_7 (F)	426(230)	484(0)	V_7 (F)
			V_7+V_9 (CB)			V_7+V_{10} (CB)
			V_7+V_{11} (CB)	426(230)		---
	361(0)	433(91)	V_6 (F)	362(0)	427(160)	V_6 (F)
			V_6+V_{54} (CB)			V_6+V_{53} (CB)
	361(0)	433(91)	V_5 (F)	362(0)	427(160)	V_5 (F)
			V_5+V_{48} (CB)			V_5+V_{53} (CB)
	361(0)	433(91)	V_4 (F)	362(0)	427(160)	V_4 (F)
			V_4+V_{53} (CB)			V_4+V_{54} (CB)
	---	365(0)	V_3 (F)	---	365(0)	V_3 (F)
			V_3+V_{15} (CB)			V_3+V_{15} (CB)
			V_3+V_{24} (CB)			V_3+V_{50} (CB)
	---	365(0)	V_2 (F)	---	365(0)	V_2 (F)
			V_2+V_{15} (CB)			V_2+V_{15} (CB)
			V_2+V_{23} (CB)			V_2+V_{51} (CB)
	---	365(0)	V_1 (F)	---	365(0)	V_1 (F)
			V_1+V_{15} (CB)			V_1+V_{52} (CB)
			V_1+V_{25} (CB)			V_1+V_{15} (CB)

Table S8. The harmonic and anharmonic infrared vibrational frequencies (in cm^{-1}) with relative intensity I (in $KM\ mol^{-1}$), including fundamental and combination modes for $C_{12}N_8$ heterofullerene in the cationic and anionic forms in the gas phase.

$C_{12}N_8$	Cation in the gas phase			Anion in the gas phase		
Vibrational Modes	Harmonic Freq (Int)	Anharmonic Freq (Int)	Mode	Harmonic Freq (Int)	Anharmonic Freq (Int)	Mode
C-C stretch	1547(0)	1354(1)	V_{51} (F)	1610(0.4)	1619(13)	V_{54} (F)
			$V_{30}+V_{51}$ (CB)			V_6+V_{54} (CB)

	1547(0)	1354(1)	V_{50} (F)		1619(13)	V_{53} (F)
			$V_{30}+V_{50}$ (CB)			$V_{3}+V_{53}$ (CB)
	1547(0)	1354(1)	V_{49} (F)			---
			$V_{29}+V_{49}$ (CB)			---
	1496(72)	1353(21)	V_{54} (F)	1604(58)	1563(5)	V_{52} (F)
			$V_{1}+V_{54}$ (CB)			$V_{5}+V_{52}$ (CB)
	1496(72)	1353(21)	V_{53} (F)			---
	1496(72)		$V_{2}+V_{53}$ (CB)			---
			$V_{3}+V_{53}$ (CB)			---
	---	1281(3)	V_{52} (F)	1549(34)	1486(1)	V_{51} (F)
			$V_{3}+V_{52}$ (CB)			$V_{2}+V_{50}$ (CB)
			$V_{49}+V_{52}$ (CB)			---
C-N stretch	1176(0)	1183(0)	V_{48} (F)	1502(73)	1468(33)	V_{50} (F)
			$V_{1}+V_{48}$ (CB)			$V_{1}+V_{51}$ (CB)
	1176(0)	1183(0)	V_{47} (F)			---
			$V_{2}+V_{47}$ (CB)			---
	1176(0)	1183(0)	V_{46} (F)			---
			$V_{43}+V_{46}$ (CB)			---
	1140(0)	1140(0)	V_{45} (F)	1489(93)	1233(15)	V_{49} (F)
			$V_{5}+V_{45}$ (CB)			$V_{49}+V_{1}$ (CB)
	1120(0)	1130(0)	V_{44} (F)	1240(45)	1222(20)	V_{48} (F)
	1120(0)		$V_{44}+V_{48}$ (CB)			$V_{48}+V_{2}$ (CB)
						$V_{48}+V_{47}$ (CB)
	1103(64)	1128(0)	V_{43} (F)	1211(77)	1192(42)	V_{47} (F)
	1103(64)		$V_{43}+V_{46}$ (CB)			$V_{4}+V_{47}$ (CB)
	1033(0)	1100(20)	V_{42} (F)	1182(0)	1157(0.5)	V_{46} (F)
			$V_{42}+V_{53}$ (CB)			$V_{2}+V_{46}$ (CB)
	1033(0)	1100(20)	V_{41} (F)			---
			$V_{41}+V_{40}$ (CB)			---
	1033(0)	1100(20)	V_{40} (F)			---
			$V_{31}+V_{40}$ (CB)			---
	967(17)	1033(0)	V_{39} (F)	1143(2)	1133(0)	V_{45} (F)
			$V_{3}+V_{39}$ (CB)			$V_{2}+V_{45}$ (CB)

967(17)	1033(0)	V_{38} (F)			---
		V_2+V_{38} (CB)			---
967(17)	1033(0)	V_{37} (F)			---
		V_1+V_{37} (CB)			---
905(0)	956(9)	V_{36} (F)	1126(0)	1102(0)	V_{44} (F)
		$V_{36}+V_{54}$ (CB)			V_5+V_{45} (CB)
905(0)	956(9)	V_{35} (F)			---
		$V_{35}+V_{53}$ (CB)			---
905(0)	956(9)	V_{34} (F)			---
		$V_{10}+V_{34}$ (CB)			---
882(0)	876(10)	V_{32} (F)	1097(0)	1071(19)	V_{43} (F)
882(0)		V_3+V_{32} (CB)			V_9+V_{43} (CB)
	868(10)	V_{33} (F)	1068(62)	1041(11)	V_{42} (F)
		V_2+V_{33} (CB)			V_9+V_{43} (CB)
	867(10)	V_{31} (F)	---		---
		V_1+V_{31} (CB)			---
778(10)	857(12)	V_{29} (F)	1039(86)	1035(14)	V_{41} (F)
778(10)		V_3+V_{29} (CB)			V_8+V_{37} (CB)
778(10)	853(14)	V_{30} (F)	1030(62)	1009(4)	V_{40} (F)
		V_1+V_{30} (CB)			V_9+V_{40} (CB)
769(0)	760(0)	V_{24} (F)	1005(5)	1000(5)	V_{39} (F)
		$V_{24}+V_{27}$ (CB)			$V_{12}+V_{39}$ (CB)
	760(0)	V_{23} (F)			---
		$V_{23}+V_{26}$ (CB)			---
	760(0)	V_{22} (F)			---
		$V_{22}+V_{28}$ (CB)			---
	752(1.1)	V_{25} (F)	985(25)	983(8)	V_{37} (F)
		V_7+V_{25} (CB)			V_8+V_{37} (CB)
			982(0)	977(0)	V_{38} (F)
					V_1+V_{38} (CB)
	---	---	976(16)	927(0)	V_{36} (F)
		---			V_1+V_{36} (CB)
	---	---	934(0)	913(0.2)	V_{35} (F)

			---			V_2+V_{35} (CB)
						V_{20}+V_{35} (CB)
		---	---	902(4)	909(0.9)	V_{34} (F)
						V_{22}+V_{34} (CB)
		---	---	899(0.04)	871(0)	V_{33} (F)
						V_{18}+V_{33} (CB)
	---	---	---	859(0)	838(4)	V_{32} (F)
						V_{23}+V_{32} (CB)
	---	---	---	833(4)	835(3)	V_{30} (F)
			---			V_{23}+V_{30} (CB)
			---			V_{29}+V_{30} (CB)
	---		---	829(23)	819(14)	V_{31} (F)
						V_6+V_{31} (CB)
CNN bend	770(0)	695(64)	V_{28} (F)	828.9(2)	796(0.5)	V_{29} (F)
			V_1+V_{28} (CB)			V_3+V_{29} (CB)
		695(64)	V_{27} (F)			---
			V_{20}+V_{27} (CB)			---
			V_{26} (F)			---
			V_{19}+V_{26} (CB)			---
	743(0)	684(1.7)	V_{21} (F)	791(0.2)	764(9)	V_{28} (F)
			V_3+V_{21} (CB)			V_1+V_{28} (CB)
	743(0)	684(1.7)	V_{20} (F)			---
			V_1+V_{20} (CB)			---
	743(0)	684(1.7)	V_{19} (F)			---
			V_2+V_{19} (CB)			---
	689(0)	627(0.2)	V_{17} (F)	764(28)	760(11)	V_{26} (F)
			V_{17}+V_{29} (CB)			V_3+V_{26} (CB)
	689(0)	627(0.2)	V_{16} (F)			V_7+V_{26} (CB)
			V_{10}+V_{16} (CB)			---
	689(0)	627(0.2)	V_{15} (F)			---
			V_{11}+V_{15} (CB)			---
	650(0)	---	---	752(3.5)	759(4)	V_{27} (F)
						V_{25}+V_{27} (CB)

625(3)	621(0)	V_{14} (F)	748(5)	736(13)	V_{24} (F)
625(3)		$V_{14}+V_{33}$ (CB)			$V_{3}+V_{24}$ (CB)
625(3)	621(0)	V_{13} (F)			---
		$V_{13}+V_{31}$ (CB)			---
613(0)	584(0)	V_{12} (F)	728(1.4)	730(0.03)	V_{25} (F)
613(0)		$V_{12}+V_{19}$ (CB)			$V_{25}+V_{27}$ (CB)
592(0)	533(2.4)	V_{18} (F)	726(24)	725(14)	V_{23} (F)
		$V_{1}+V_{18}$ (CB)			$V_{4}+V_{23}$ (CB)
					$V_{23}+V_{28}$ (CB)
499(0)	444(189)	V_{7} (F)	717(10)	713(0)	V_{22} (F)
		$V_{7}+V_{25}$ (CB)			$V_{22}+V_{34}$ (CB)
499(0)	444(189)	V_{8} (F)			
		$V_{8}+V_{18}$ (CB)			
	444(189)	V_{9} (F)			
		$V_{9}+V_{18}$ (CB)			
---	---	---	704(0)	706(9)	V_{20} (F)
					$V_{4}+V_{20}$ (CB)
					$V_{11}+V_{20}$ (CB)
---	---	---	698.6(4)	703(14)	V_{21} (F)
---	---	---	698.6(4)		$V_{21}+V_{32}$ (CB)
---	---	---	691(2)	701(0.2)	V_{19} (F)
					$V_{13}+V_{19}$ (CB)
			689(0)	701(0.2)	V_{18} (F)
					$V_{18}+V_{33}$ (CB)
			674(62)	672(44)	V_{17} (F)
					$V_{17}+V_{31}$ (CB)
---	---	---	631(12)	630(5)	V_{16} (F)
					$V_{6}+V_{16}$ (CB)
---	---	---	600(0)	600(0)	V_{15} (F)
					$V_{6}+V_{15}$ (CB)
---	---	---	598(10)	588(0)	V_{13} (F)
					$V_{13}+V_{14}$ (CB)
---	---	---	584(0)	585(6)	V_{14} (F)

						$V_{14}+V_{49}$ (CB)
Ring distort	490(260)	427(0)	V_{11} (F)	555(4)	572(0.3)	V_{11} (F)
	490(260)	---	V_2+V_{11} (CB)			$V_{11}+V_{20}$ (CB)
			$V_{11}+V_{17}$ (CB)			---
	338(0)	281(0)	V_{10} (F)	554(0.003)	556(18)	V_{12} (F)
	338(0)		$V_{10}+V_{16}$ (CB)			$V_{12}+V_{39}$ (CB)
	338(0)					
	281(4)	153(0)	V_6 (F)	537(0)	539(0)	V_{10} (F)
			V_2+V_6 (CB)			$V_{10}+V_2$ (CB)
	281(4)	153(0)	V_5 (F)			V_4+V_{10} (CB)
			V_3+V_5 (CB)			---
	281(4)	153(0)	V_4 (F)			---
			V_4+V_{26} (CB)			---
			V_1+V_4 (CB)			---
	---	11.35(7)	V_2 (F)	532(10)	537(12)	V_9 (F)
	---	---	V_2+V_{53} (CB)			V_9+V_2 (CB)
		11.35(7)	V_1 (F)			---
			V_1+V_{54} (CB)			---
	---	10.44(7)	V_3 (F)	502(13)	504(10)	V_8 (F)
		---	V_3+V_{29} (CB)			V_8+V_{37} (CB)
				461(0.04)	454(0.01)	V_7 (F)
	---	---	---			V_7+V_{26} (CB)
				416(170)	406(166)	V_6 (F)
						V_6+V_{16} (CB)
	---	---	---	405(203)	395(172)	V_5 (F)
	---	---	---			V_4+V_5 (CB)
			---	319(52)	306(70)	V_4 (F)
			---			V_4+V_5 (CB)
	---	---	---			V_4+V_{23} (CB)
	---	---	---	290(0)	285(0)	V_3 (F)
						V_3+V_6 (CB)
						V_3+V_{19} (CB)
						V_3+V_{26} (CB)

---	---	---	288(136)	263(125)	V_2 (F)
					V_1+V_2 (CB)
---	---	---	271(2)	261(2)	V_1 (F)
					V_1+V_2 (CB)
					V_1+V_{28} (CB)
					V_1+V_{49} (CB)
